# Supplementary material for: CtBP1-Mediated Membrane Fission Contributes to Effective Recycling of Synaptic Vesicles
Source: Cell Rep. 2020 Feb 18;30(7):2444–2459.e7. doi: 10.1016/j.celrep.2020.01.079 (PMC7034063; doi:10.1016/j.celrep.2020.01.079)
Supplement: Document S1. Figures S1–S5 and Tables S1 and S2 [file mmc1.pdf]

Cell Reports, Volume *30*

Supplemental Information

CtBP1-Mediated Membrane Fission Contributes to Effective Recycling of Synaptic Vesicles

Daniela Ivanova, Cordelia Imig, Marcial Camacho, Annika Reinhold, Debarpan Guhathakurta, Carolina Montenegro-Venegas, Michael A. Cousin, Eckart D. Gundelfinger, Christian Rosenmund, Benjamin Cooper, and Anna Fejtova

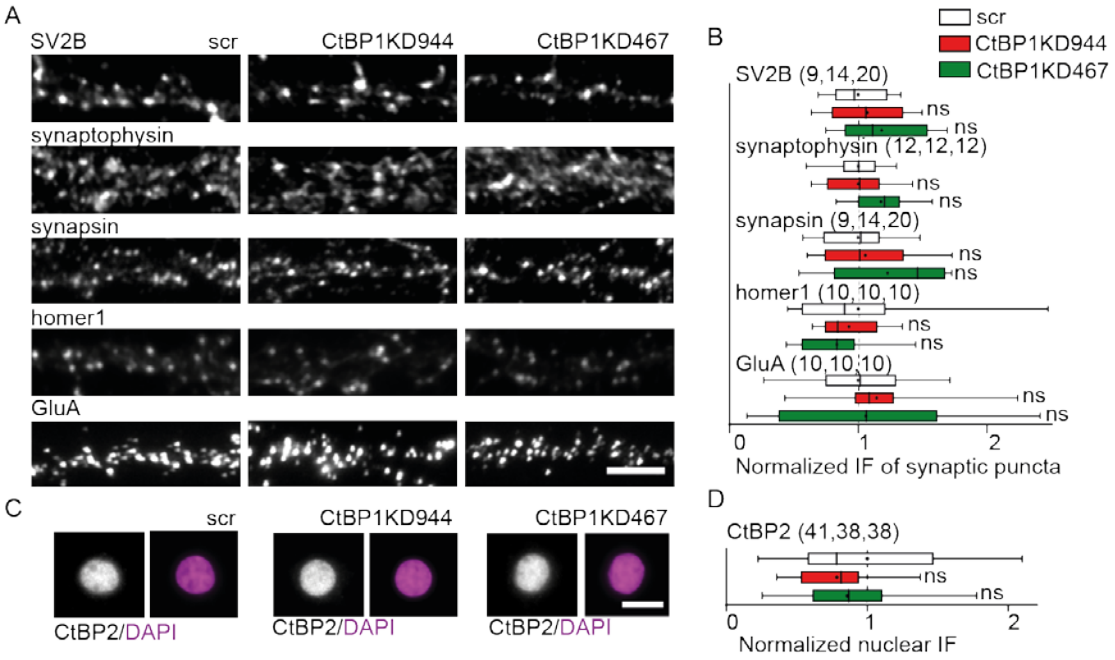

Figure S1 (Related to Figure 1)

Knock down of CtBP1 does not affect the overall expression of synaptic proteins and CtBP2

A) Synaptic abundance of pre- (SV2B, synapsin, synaptophysin) and post-synaptic markers (homer1, GluA) does not change in CtBP1KD neurons.
B) Quantification of the effects shown in A)
C) Nuclear CtBP2 does not change in CtBP1KD neurons.
D) Quantification of the effects shown in C)

Scale bar is 5 µm in A, and 10 µm in C. In the plots the interquartile range and median are depicted as boxes, minimal and maximal values as whiskers and + indicates mean.

Figure S2

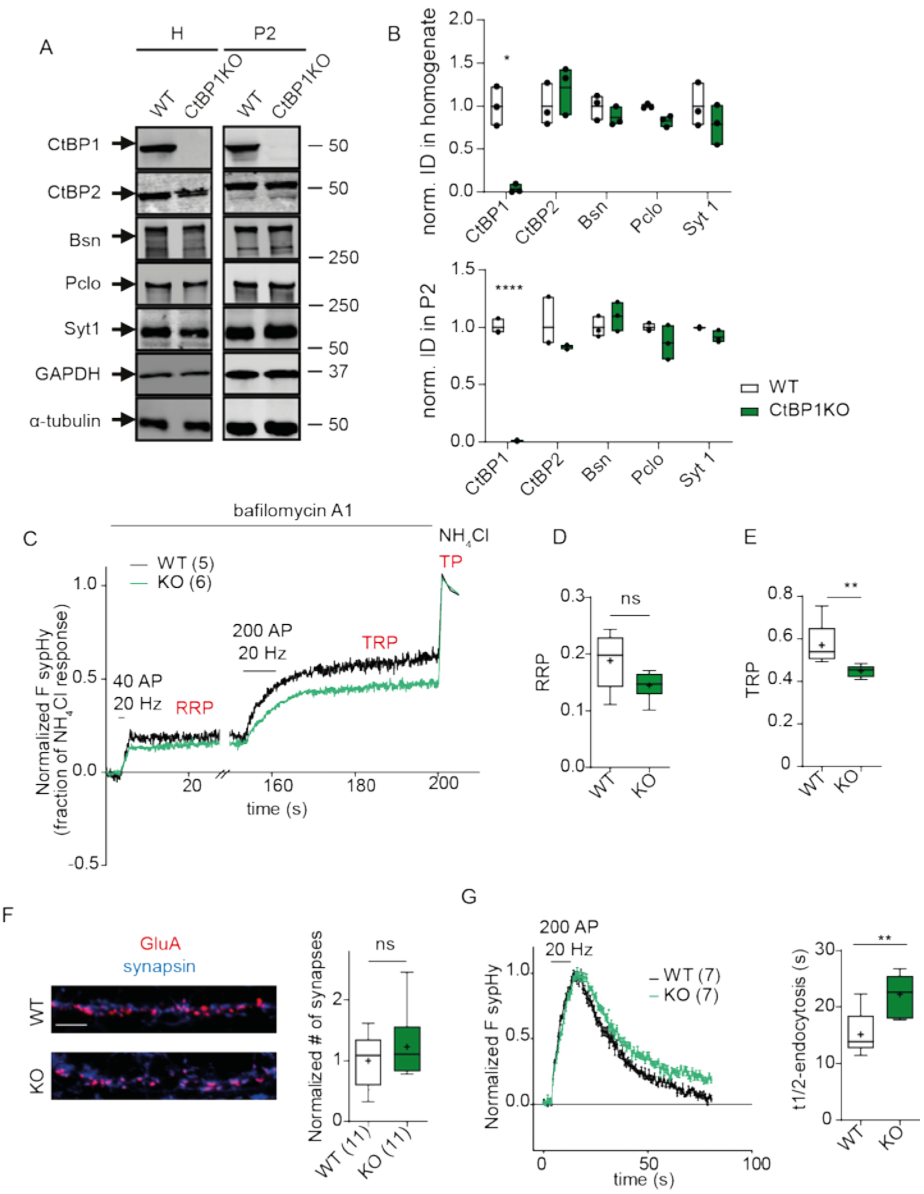

Figure S2 (Related to Figure 1)

***Ctbp1* KO synapses have a reduced rate of SV endocytosis and a lower number of release-competent vesicles.**

A) Immunoblot detection of synaptic proteins in brain homogenates (H) and crude synaptosomes (P2) from WT and *CtBP1*-/- mice. GAPDH and α-tubulin are loading controls.

B) Quantification of the effects shown in A)

C) Average sypHy-fluorescence traces reporting SV pool sizes from neurons derived from WT and *Ctbp1*-/- mice.

D) The mean values of RRP in WT and *Ctbp1*-/- did not differ significantly.

E) Quantification of TRP size in WT and *Ctbp1*-/- .

F) Neurons prepared from *Ctbp1*-/- animals and their WT siblings stained with an anti synapsin Ab, to label presynaptic terminals and pan anti GluA Ab to label postsynapses. Number of co-localizing synapsin and GluA puncta was slightly but not significantly increased in KO compared to control. The overlays are shown in the indicated colors. Scale bar: 5µm.

G) Peak-normalized sypHy responses to 200 AP at 20Hz. The half times: t1/2 of endocytosis (bar graph) were smaller in WT neurons compared to *Ctbp1*-/- .

In the plots the interquartile range and median are depicted as boxes, minimal and maximal values as whiskers and + indicates mean. Significance is indicated using asterisks: nsP > 0.05, ∗p < 0.05, ∗∗p < 0.01, ∗∗∗∗ p < 0.0001.

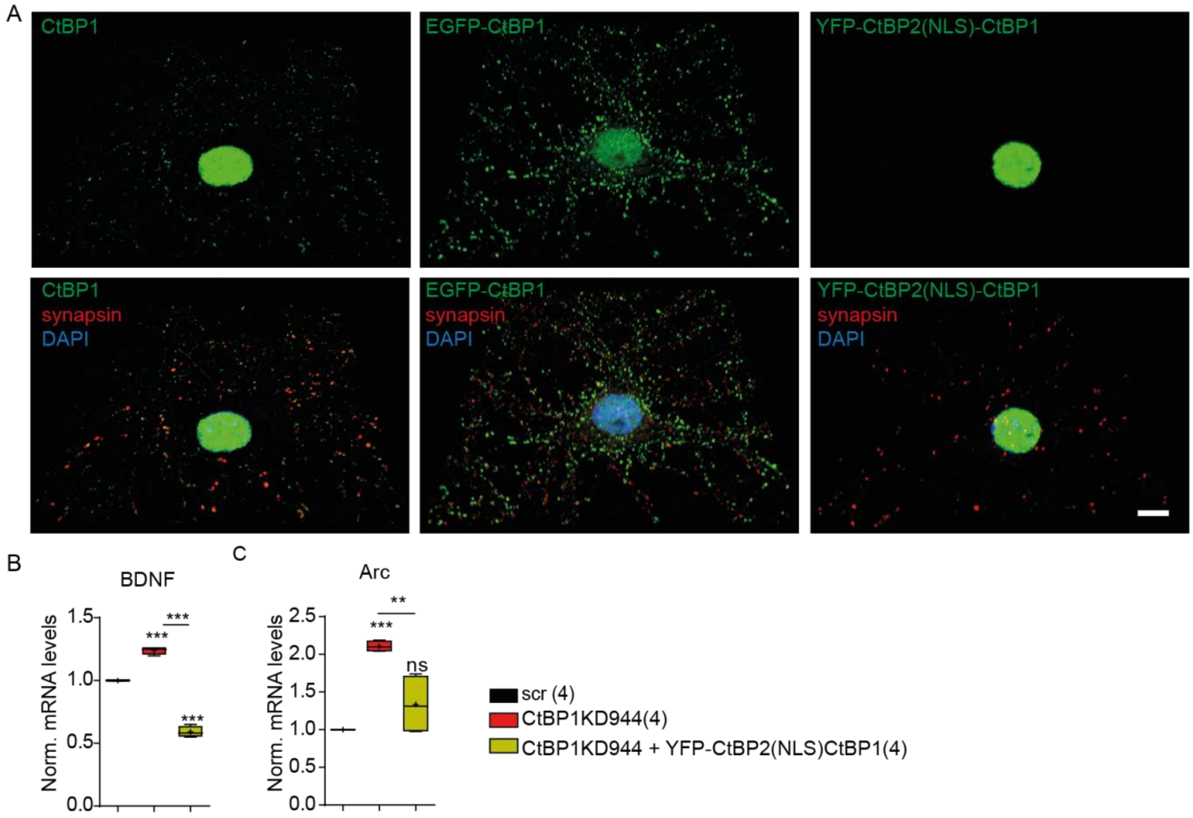

Figure S3 (Related to Figure 3 and 4)

Expression of YFP-CtBP2(NLS)-CtBP1 reverts the effect of CtBP1KD944 on gene expression.

A) Perspective views of 3D reconstructions of hippocampal neurons showing the synapto-nuclear distribution of the endogenous CtBP1 and the expressed rescue variants. Synapsin staining labels presynaptic terminals; DAPI labels nuclei. Note that EGFP-CtBP1 shows a decreased nuclear and an increased synaptic localization, whereas YFP-CtBP2(NLS)-CtBP1 is expressed only in the nucleus. For better visualization several EGFP-CtBP1-positive spots were removed from the planes above the nucleus. Overlays are shown in the indicated colors. Scale bar: 7µm.

B and C) YFP-CtBP2(NLS)-CtBP1 counteracts the increased expression of BDNF and Arc in CtBP1KD944 neuronal cultures.

In the plots the interquartile range and median are depicted as boxes, minimal and maximal values as whiskers and + indicates mean. In the graphs comparisons with the control are indicated above each box and, comparisons between the conditions are given as horizontal bars. Significance is indicated using asterisks: ∗∗p < 0.01, ∗∗∗p < 0.001.

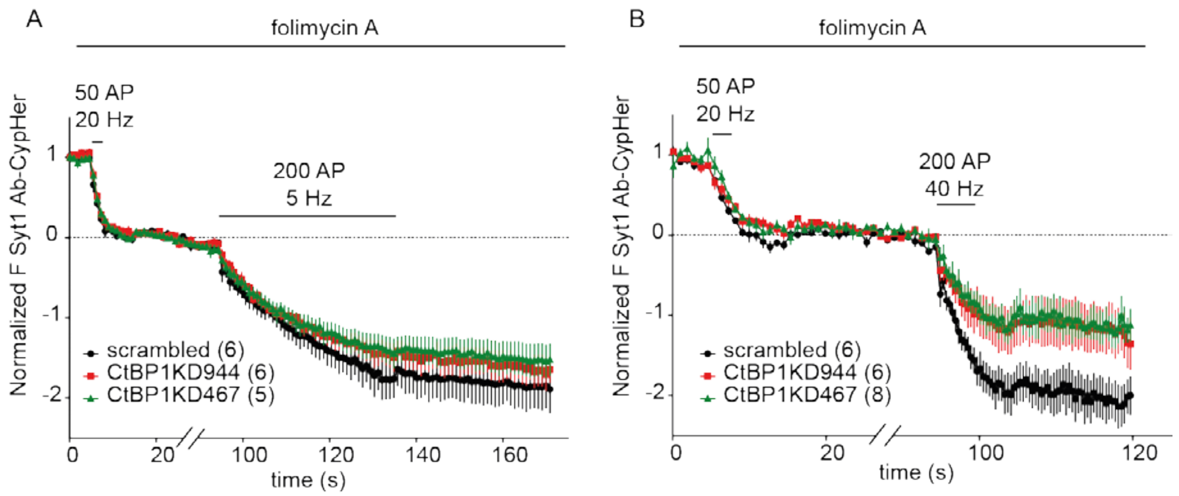

Figure S4 (Related to Figure 4)

Frequency-dependent short-term synaptic depression at CtBP1-deficient synapses

A) and B) Average Syt1 Ab-CypHer responses to 50 AP at 20 Hz (a reference response), followed by a 60s rest period and 200 AP at 5 Hz (A) or 40 Hz (B) in the presence of 80 nM folimycin. The traces were normalized to the amplitudes of the reference response. KD of CtBP1 reduces the fluorescence responses to 200 AP at 5 Hz and even more pronouncedly at 40 Hz.

Data points in curves are depicted as means and SEM.

Figure S5

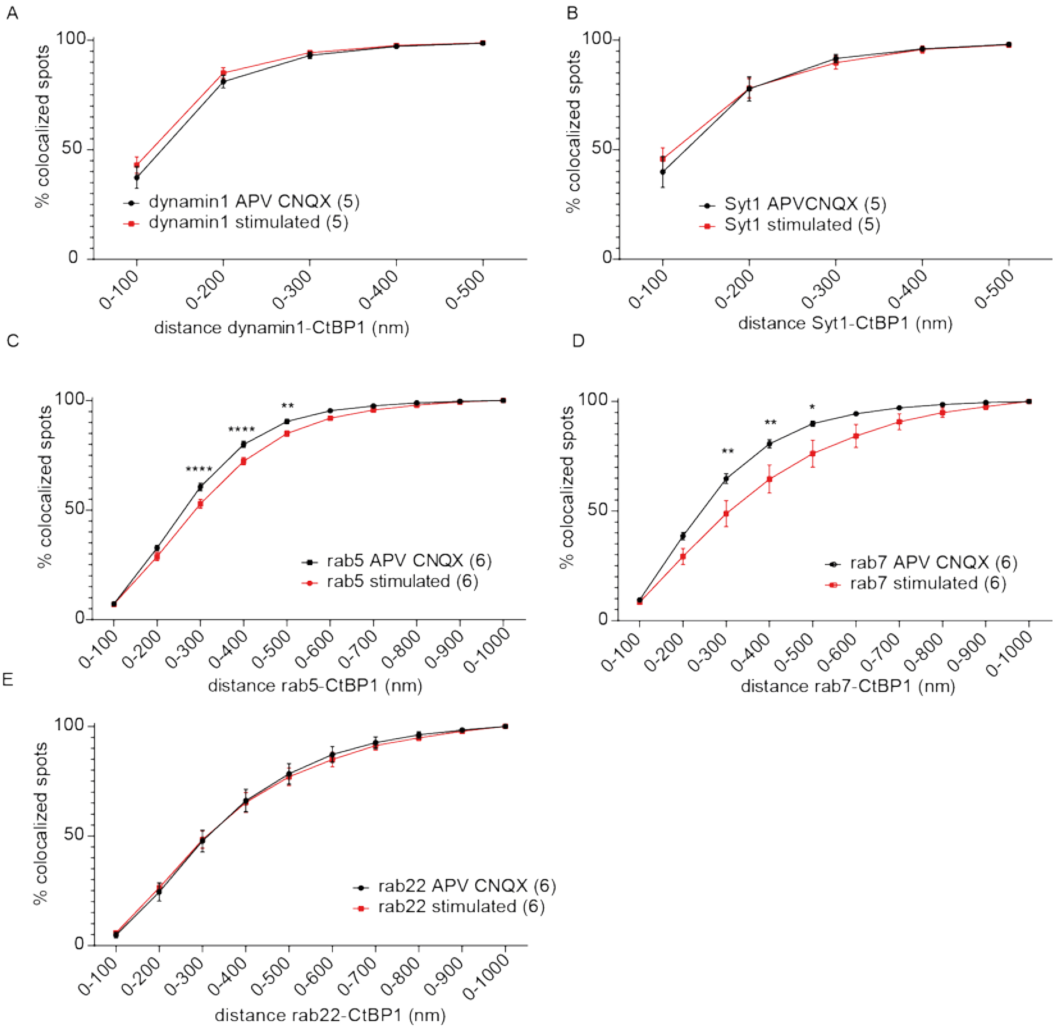

Figure S5 (Related to Figure 5)

Effect of synaptic stimulation on the co-localization of CtBP1 with the endocytic markers dynamin1, rab5, rab7, rab22 and the SV protein Syt1.

A - E) Cumulative plots showing the % of dynamin1, rab5, rab7, rab22 and Syt1 puncta co-localizing with CtBP1 in control (treated with 50µM APV and 10µM CNQX for 10 min) and stimulated (200AP at 40Hz) neurons, binned according to the distance to CtBP1 labelled spots.

Data points are depicted as means and SEM. Significance is indicated using asterisks: ∗p < 0.05, ∗∗p < 0.01, ∗∗∗∗ p < 0.0001.

Table S1: Quantitative analysis of synaptic ultrastructure (Related to Figure 2)

2D EM Analysis of Synaptic Morphology

	**WT (N=3, n=159)**	**KO (N=4, n=146)**	
# of SVs per profile	80.72 ± 3.244	89.21 ± 3.721	P = 0.098
terminal area (x 0.01 µm^2)	40.38 ± 1.182	41.19 ± 1.303	P = 0.845
# SVs / 0.01 µm^2 terminal area	1.993 ± 0.054	2.159 ± 0.064	P = 0.065
PSD length (nm)	373.7 ± 9.261	379.4 ± 9.421	P = 0.627
# of endosomes / terminal	0.843 ± 0.077	0.726 ± 0.082	P = 0.140
# of LDCVs / terminal	0.151 ± 0.034	0.24 ± 0.043	P = 0.083

N, number of animals; n, number of synaptic profiles; SV, synaptic vesicle; PSD, postsynaptic density; LDCV, large dense-core vesicle. (red P-values = Mann-Whitney test, black P-values = unpaired t-test)

3D Electron Tomographic Analysis of Synaptic Vesicle Pools

	**WT (N=3, n=26)**	**KO (N=4, n=25)**	
# SVs within 0-2 nm of AZ	0.605 ± 0.092	0.876 ± 0.117	P = 0.075
# SVs within 0-5 nm of AZ	0.797 ± 0.109	1.213 ± 0.142	*P = 0.043
# SVs within 0-40 nm of AZ	1.821 ± 0.12	2.496 ± 0.168	**P = 0.002
# SVs within 0-100 nm of AZ	5.876 ± 0.267	7.307 ± 0.382	**P = 0.003
# SVs within 0-200 nm of AZ	14.65 ± 0.817	15.31 ± 0.811	P = 0.572
# SVs within 5-10 nm of AZ	0.214 ± 0.041	0.292 ± 0.07	P = 0.621
# SVs within 10-20 nm of AZ	0.264 ± 0.058	0.162 ± 0.037	P = 0.354
# SVs within 20-30 nm of AZ	0.213 ± 0.051	0.363 ± 0.069	P = 0.072
# SVs within 30-40 nm of AZ	0.345 ± 0.052	0.465 ± 0.07	P = 0.170
# SVs within 40-50 nm of AZ	0.531 ± 0.053	0.596 ± 0.081	P = 0.503
# SVs within 50-100 nm of AZ	3.54 ± 0.196	4.215 ± 0.245	*P = 0.036
# SVs within 100-150 nm of AZ	4.408 ± 0.331	4.175 ± 0.251	P = 0.579
# SVs within 150-200 nm of AZ	4.34 ± 0.328	3.827 ± 0.291	P = 0.249
AZ area (nm^2)	40.900 ± 1.775	44.240 ± 2.276	P = 0.569
SV diameter (SVs within 0-200 nm of AZ)	44.95 ± 0.347	45.77 ± 0.38	P = 0.114
SV diameter (SVs within 0-100 nm of AZ)	44.98 ± 0.381	45.82 ± 0.426	P = 0.15

N, number of animals; n, number of tomograms; SV, synaptic vesicle; AZ, active zone. SV numbers within a certain distance of the AZ are normalized to 0.01 µm^2 of AZ area. Values indicate mean ± SEM. (red P-values = Mann-Whitney test, black P-values = unpaired t-test)

	**WT (n=63)**	**KO (n=100)**	
SV diameter (docked SVs, 0-2 nm of AZ)	44.17 ± 0.64	46.08 ± 0.485	*P = 0.012

n, number of docked SVs averaged over all tomograms of a given genotype

Table S2: Electrophysiological analysis of autaptic cultures from CtBP1944KD and scr and upon expression of selective synaptic or nuclear rescue constructs (Related to Figure 3)

<table>
<tr><th></th><th>SC</th><th>Kruskal-Wallis test</th><th>CtBP1KD944</th><th>Kruskal-Wallis test</th><th>EGFP-CtBP1</th><th>Kruskal-Wallis test</th><th>YFP-CtBP2(NLS)-CtBP1</th><th>Kruskal-Wallis test</th></tr>
<tr><td rowspan="3">mEPSC charge (fC)</td><td rowspan="3">110.5 ± 4.2 (n=69/5)</td><td>CtBP1KD944 P>0.99</td><td rowspan="3">104.4 ± 4.1 (n=70/5)</td><td>SC P>0.99</td><td rowspan="3">119.4 ± 9.8 (n=64/5)</td><td>SC P>0.99</td><td rowspan="3">110.3 ± 4.1 (n=62/5)</td><td>SC P>0.99</td></tr>
<tr><td>EGFP-CtBP1 P>0.99</td><td>EGFP-CtBP1 P>0.99</td><td>CtBP1KD944 P>0.99</td><td>CtBP1KD944 P>0.99</td></tr>
<tr><td>YFP-CtBP2(NLS)-CtBP1 P>0.99</td><td>YFP-CtBP2(NLS)-CtBP1 P>0.99</td><td>YFP-CtBP2(NLS)-CtBP1 P>0.99</td><td>EGFP-CtBP1 P>0.99</td></tr>
</table>

EPSC Charge (pC)	35.4 ± 4.5 (n=77/5)	CtBP1KD944 P=0.0018	55.2 ± 5.9 (n=72/5)	SC P=0.0018	78.1 ± 8.5 (n=62/5)	SC <0.0001	51.3 ± 6.2 (n=63/5)	SC P=0.072
		EGFP-CtBP1 P<0.0001		EGFP-CtBP1 P=0.4137		CtBP1KD944 P=0.4137		CtBP1KD944 P>0.99
		YFP-CtBP2(NLS)-CtBP1 P=0.072		YFP-CtBP2(NLS)-CtBP1 P>0.99		YFP-CtBP2(NLS)-CtBP1 P=0.0436		EGFP-CtBP1 P=0.0436
Pvr (%)	7.0 ± 0.5 (n=73/5)	CtBP1KD944 P<0.0001	15.8 ± 0.9 (n=64/5)	SC P<0.0001	14.2 ± 1.1 (n=52/5)	SC P<0.0001	11.6 ± 1.0 (n=62/5)	SC P>0.006
		EGFP-CtBP1 P<0.0001		EGFP-CtBP1 P>0.999		CtBP1KD944 P>0.999		CtBP1KD944 P=0.011
		YFP-CtBP2(NLS)-CtBP1 P>0.006		YFP-CtBP2(NLS)-CtBP1 P=0.011		YFP-CtBP2(NLS)-CtBP1 P=0.1925		EGFP-CtBP1 P=0.1925

n, number of neurons / independent cultures analyzed
