# Supplementary material for: CtBP1-Mediated Membrane Fission Contributes to Effective Recycling of Synaptic Vesicles
Source: Cell Rep. 2020 Feb 18;30(7):2444–2459.e7. doi: 10.1016/j.celrep.2020.01.079 (PMC7034063; doi:10.1016/j.celrep.2020.01.079)
Supplement: Document S2. Article plus Supplemental Information [file mmc3.pdf]

Cell Reports

Article

CtBP1-Mediated Membrane Fission Contributes to Effective Recycling of Synaptic Vesicles

Graphical Abstract

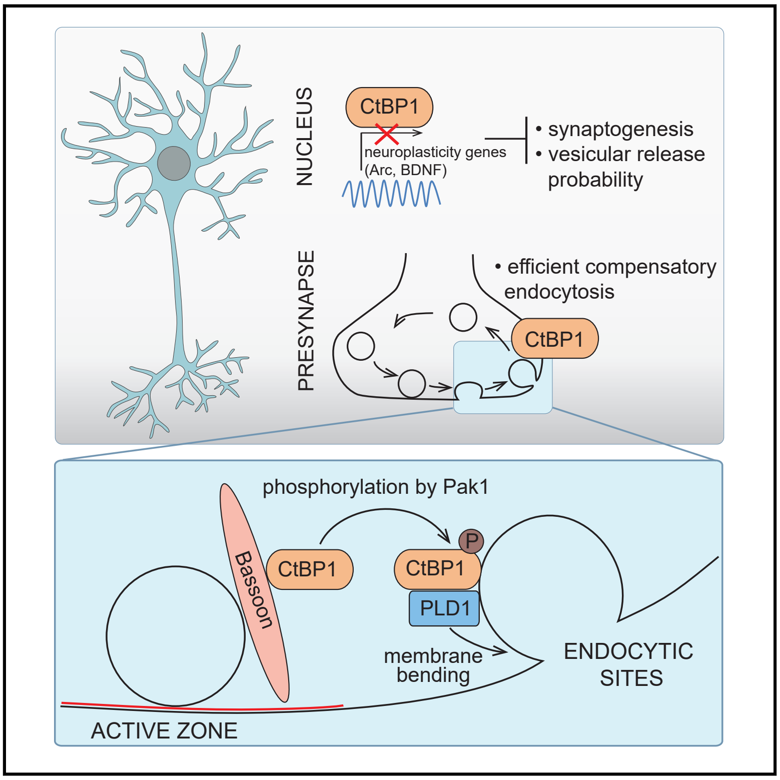

Highlights

- Nuclear CtBP1 restricts synaptogenesis and vesicular release probability
- CtBP1 is required for efficient compensatory endocytosis at the presynapse
- CtBP1 facilitates synaptic vesicle retrieval via activation of the lipid enzyme PLD1
- Phosphorylation by Pak1 shifts CtBP1 from the active zone to endocytic sites

Authors

Daniela Ivanova, Cordelia Imig, Marcial Camacho, ..., Christian Rosenmund, Benjamin Cooper, Anna Fejtova

Correspondence

anna.fejtova@uk-erlangen.de

In Brief

Ivanova et al. demonstrate a dual role of CtBP1 in synaptic transmission. Nuclear CtBP1 restricts synaptogenesis and vesicular release probability, whereas presynaptic CtBP1 promotes compensatory endocytosis via activation of the lipid enzyme PLD1. Phosphorylation by Pak1 controls the redistribution of CtBP1 from active zones toward endocytic sites linking presynaptic exo- and endocytosis.

Ivanova et al., 2020, Cell Reports *30*, 2444–2459
February 18, 2020 © 2020 The Authors.
https://doi.org/10.1016/j.celrep.2020.01.079

OPEN ACCESS
CellPress

Cell Reports
Article

CtBP1-Mediated Membrane Fission Contributes to Effective Recycling of Synaptic Vesicles

Daniela Ivanova,[1,2,3,8] Cordelia Imig,[4,9] Marcial Camacho,[5,9] Annika Reinhold,[5] Debarpan Guhathakurta,[3] Carolina Montenegro-Venegas,[2] Michael A. Cousin,[6] Eckart D. Gundelfinger,[2,7] Christian Rosenmund,[5] Benjamin Cooper,[4] and Anna Fejtova[1,2,3,10,*]
[1]RG Presynaptic Plasticity, Leibniz Institute for Neurobiology, Magdeburg, Germany
[2]Department of Neurochemistry and Molecular Biology, Leibniz Institute for Neurobiology, Magdeburg, Germany
[3]Molecular Psychiatry, Department of Psychiatry and Psychotherapy, University Hospital Erlangen, Friedrich-Alexander-Universität Erlangen-Nürnberg (FAU), Erlangen, Germany
[4]Department of Molecular Neurobiology, Max Planck Institute of Experimental Medicine, 37075 Göttingen, German
[5]Institute of Neurophysiology, Charité-Universitätsmedizin Berlin, Berlin, Germany
[6]Centre for Discovery Brain Sciences, Hugh Robson Building, George Square, University of Edinburgh, EH9 9XD Edinburgh, UK
[7]Center for Behavioral Brain Science and Medical Faculty, Otto von Guericke University Magdeburg, Magdeburg, Germany
[8]Present address: Centre for Discovery Brain Sciences, Hugh Robson Building, George Square, University of Edinburgh, EH9 9XD Edinburgh, UK
[9]These authors contributed equally
[10]Lead Contact
*Correspondence: anna.fejtova@uk-erlangen.de
https://doi.org/10.1016/j.celrep.2020.01.079

SUMMARY

Compensatory endocytosis of released synaptic vesicles (SVs) relies on coordinated signaling at the lipid-protein interface. Here, we address the synaptic function of C-terminal binding protein 1 (CtBP1), a ubiquitous regulator of gene expression and membrane trafficking in cultured hippocampal neurons. In the absence of CtBP1, synapses form in greater density and show changes in SV distribution and size. The increased basal neurotransmission and enhanced synaptic depression could be attributed to a higher vesicular release probability and a smaller fraction of release-competent SVs, respectively. Rescue experiments with specifically targeted constructs indicate that, while synaptogenesis and release probability are controlled by nuclear CtBP1, the efficient recycling of SVs relies on its synaptic expression. The ability of presynaptic CtBP1 to facilitate compensatory endocytosis depends on its membrane-fission activity and the activation of the lipid-metabolizing enzyme PLD1. Thus, CtBP1 regulates SV recycling by promoting a permissive lipid environment for compensatory endocytosis.

INTRODUCTION

C-terminal binding protein 1 (CtBP1) is a ubiquitously expressed dual-function protein that acts as a transcriptional corepressor in the cell nucleus and as a regulator of membrane fission in the cytoplasm (Chinnadurai, 2009; Valente et al., 2013). It is expressed in most types of neurons, where it shows a distinct localization to nuclei and presynapses (Hübler et al., 2012; tom Dieck et al., 2005). Presynaptic CtBP1 is localized near the active zone via its direct binding to two large, highly homologous active zone scaffolding proteins: bassoon (Bsn) and piccolo (Pclo) (Ivanova et al., 2015; tom Dieck et al., 2005). A dynamic synapto-nuclear shuttling of CtBP1, induced by changes in its affinity to Bsn and regulated by neuronal activity and cellular NAD/NADH ratio was shown to control the expression of a variety of neuroplasticity-related genes (Ivanova et al., 2015, 2016). Although the importance of CtBP1-dependent transcriptional regulation of neuroplasticity genes emerged from recent studies (Garriga-Canut et al., 2006; Ivanova et al., 2015, 2016), the role of synaptic CtBP1 is still elusive. Here, we hypothesize that, in addition to being implicated in the remote control of gene expression, synaptic CtBP1 might directly contribute to neurotransmitter release and SV recycling. The involvement of CtBP1 in various membrane fission processes at the Golgi and plasma membrane in non-neuronal cells is in support of this view (Valente et al., 2013). Although the mechanism of CtBP1-mediated fission remains controversial, an increasing body of evidence suggests that it induces formation of vesicular carriers by recruiting and orchestrating numerous enzymes that promote local lipid reorganization leading to membrane bending (Valente et al., 2013). This is mechanistically distinct from the principle of torsional force used in dynamin-mediated fission, most commonly implied in SV recycling (Antonny et al., 2016; Renard et al., 2018). Despite the well-established role of dynamin in SV fission, recent findings suggest that dynamin-independent forms of endocytosis might occur at hippocampal synapses (Gan and Watanabe, 2018; Wu et al., 2014). Moreover, a crosstalk and cooperativity between dynamin-mediated fission, actin cytoskeleton-mediated vesicle reformation and lipid reorganization by lipid-modifying enzymes in the execution of SV recycling were recently suggested (Puchkov and Haucke, 2013; Soykan et al., 2017; Wu et al., 2016).

In this study, we investigated the potential role of synaptic CtBP1 in the regulation of SV fusion and recycling. Using knockdown (KD), knockout (KO), and complementation approaches,

This is an open access article under the CC BY license (http://creativecommons.org/licenses/by/4.0/).

OPEN ACCESS
CellPress

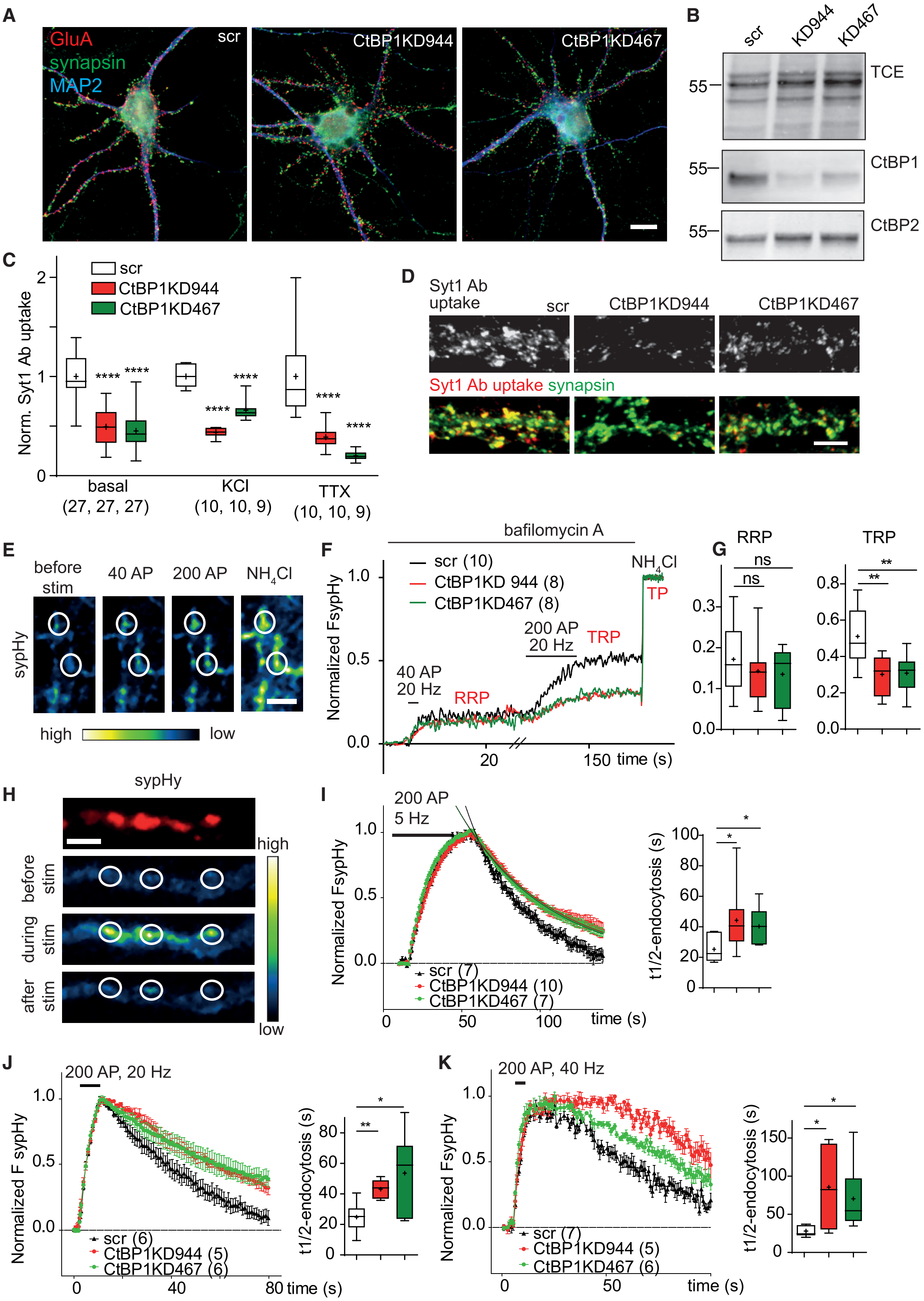

(legend on next page)

we demonstrate that loss of nuclear CtBP1 expression increases synaptogenesis and the release probability of SVs, whereas the depletion of synaptic CtBP1 leads to defects in SV retrieval, accompanied by an enlargement of the docked synaptic vesicles and pronounced synaptic depression during sustained neurotransmission. Functional experiments and super-resolution imaging indicate that synaptic CtBP1 acts at the same membrane domain as dynamin to promote SV recycling. Our results revealed a crucial requirement for CtBP1-mediated membrane fission and the activity of Phospholipase D1 (PLD1) in this process. Finally, we show that CtBP1 phosphorylation by the signaling kinase p21 (RAC1) activated kinase 1 (Pak1) provides a molecular switch controlling its re-distribution from the active zone protein Bsn to the endocytic effector PLD1, thus fine tuning its membrane trafficking activity and potentially linking presynaptic exo- and endocytic processes.

RESULTS

CtBP1 Contributes to Synaptic Vesicle Retrieval and Regulates the Size of the Total Recycling Pool

To assess whether the absence of CtBP1 affects synaptic structure and function, we used a previously established RNA-interference approach in cultured hippocampal neurons (Ivanova et al., 2015). Significant downregulation of CtBP1, but no obvious differences in the morphology and the expression of pre- and post-synaptic markers or CtBP2, a close homolog of CtBP1, were observed between controls expressing scrambled short hairpin RNA (shRNA) (scr) and CtBP1 knockdown (CtBP1KD) neurons expressing target shRNAs: CtBP1KD944 or CtBP1KD467 (Figures 1A, 1B, and S1A–S1D). Likewise, no regulation of synaptic proteins and CtBP2 was observed in homogenates or P2 fractions obtained from brains of *CtBP1* knockout animals (Figures S2A and S2B). To assess SV turnover in the absence of CtBP1, we applied a fluorophore-coupled antibody recognizing the lumenal domain of the integral SV protein synaptotagmin 1 (Syt1 Ab) to living neurons. Syt1 Ab binds to its epitope, which is transiently accessible upon SV fusion with the plasma membrane until its internalization during compensatory endocytosis. The fluorescence intensity of the internalized Syt1 Ab provides an estimate of SV recycling at individual synapses (Kraszewski et al., 1995; Lazarevic et al., 2011). The Syt1 Ab uptake driven by endogenous activity (network activity-driven release) was reduced by about 50% in CtBP1KD neurons as compared with controls (30-min incubation; Figures 1C and 1D). To address the potential contribution of an increased neuronal network activity to this phenotype and isolate presynaptic effects, we also measured the spontaneous (i.e., action-potential-independent) SV recycling within 30 min in the presence of TTX and the pool of all fusion-competent vesicles (total recycling pool [TRP]) upon brief depolarization with 50 mM KCl. In both conditions, Syt1 Ab uptake was strongly reduced (~50%) in CtBP1KD (Figure 1C), indicating an impairment in both evoked and spontaneous SV recycling at CtBP1-deficient synapses.

To monitor SV recycling by an alternative approach, we expressed scr and CtBP1KD944 and CtBP1KD467 from a bicistronic vector together with ratio-sypHy (sypHy) (Figure 1E). SypHy is an indicator composed of the SV protein synaptophysin 1 fused to pH-sensitive GFP in one of the luminal domains and tdimer 2 in the cytoplasmic domain, which allows its visualization before stimulation (Granseth et al., 2006; Rose et al., 2013). The fluorescence of sypHy increases upon SV exocytosis and decays after SV endocytosis and re-acidification. To determine the sizes of the readily releasable pool (RRP) and the recycling pool (RP), we used bafilomycin A1, a blocker of the vesicular proton pump that prevents the re-acidification of endocytosed SVs and thus the decline of sypHy fluorescence (Burrone et al., 2006). Exocytosis of the SVs from RRP and RP was evoked by the sequential delivery of 40 and 200 action potentials (APs) at 20 Hz (Figures 1E–1G). In CtBP1KD neurons, around 14% of the sypHy-positive SVs fused upon stimulation with 40 APs at 20 Hz (i.e., RRP), which was comparable to control neurons. The delivery of an additional 200 APs triggered exocytosis of ~50% of all sypHy-labeled SVs in controls, but only ~30% in CtBP1KD neurons, indicating a role of CtBP1 in the control of TRP (comprising RRP and RP). Alkalization with ammonium chloride, which de-quenches all

Figure 1. KD of CtBP1 Reduces SV Recycling

(A) Representative images showing that the general neuronal morphology and the localization of synaptic markers are not changed in CtBP1KD neurons.

(B) Representative western blots of samples from rat neurons transduced with viruses expressing shRNAs: scr, CtBP1KD944, and KD467, together with sypHy. The immunoreactivity for CtBP1 and CtBP2 and TCE total protein stain used as a loading control are shown. Although a notable downregulation of CtBP1 is evident in KD samples compared with scr, no changes were detected for CtBP2.

(C) Quantification of the Syt1 Ab uptake, driven by basal network activity, depolarization with 50 mM KCl, or in the presence of 1 μM TTX in scr, and KD cultures.

(D) Representative images of Syt1 Ab uptake, driven by basal neuronal network activity in control (scr) and CtBP1KD944 and CtBP1KD467 cultures.

(E) Representative images of neurons expressing sypHy used to determine SV pool sizes. Cells were imaged in the presence of bafilomycin A1 during stimulation with 40 APs at 20 Hz to release RRP. After a rest for 2 min, a train of 200 APs at 20 Hz triggered the exocytosis of all release-competent vesicles (TRP). A final NH_4Cl pulse, which visualized all released and non-released sypHy-positive vesicles (total pool: TP), was used for normalization.

(F) Average sypHy-fluorescence (FsypHy) traces reporting SV pool sizes from control and CtBP1KD neurons. RRP and TRP are given as fractions of TP.

(G) The mean values of RRP in scr, CtBP1KD944, and CtBP1KD467 did not differ significantly, but the KD of CtBP1 led to a significant reduction in TRP size.

(H) Images of sypHy showing SV exo-endocytosis at synapses in response to 200 APs at 5 Hz. The top image shows the reference (F) of tdimer 2 before stimulation and the bottom three panels show the green (F) of sypHy before, during, and after the stimulation.

(I–K) CtBP1 depletion results in slower retrieval of exocytosed SV. Peak-normalized sypHy responses to 200 APs at 5 Hz (I), 200 APs at 20 Hz (J), and 200 APs at 40 Hz (K), and respective single exponential fits of fluorescence decay are shown for each group. The estimated half times of endocytosis ($t_{1/2}$) are plotted. Overlays are shown in the indicated colors. Scale bars: 10 μm in (A); 5 μm in (D), (E), and (H).

In the plots the interquartile range and median are depicted as boxes, minimal and maximal values as whiskers and + indicates mean. In the graphs comparisons with the control are indicated above each box and, comparisons between the conditions are given as horizontal bars. Significance is indicated using asterisks: nsP > 0.05, *p < 0.05, **p < 0.01, ***p < 0.001, **** p < 0.0001.

sypHy-positive SVs, revealed no differences in its expression between CtBP1KD and control neurons. (Figures 1E–1G) An analogous analysis performed in cultured neurons isolated from constitutive *Ctbp1* KO mice recapitulated the results of the KD approach and confirmed the significant reduction of TRP in CtBP1-deficient synapses (Figures S2C–S2E).

To assess potential changes in the kinetics of SV exo-endocytosis in the absence of CtBP1, we monitored sypHy responses evoked by a train of 200 APs at 5, 20, or 40 Hz in neurons expressing CtBP1KD944, CtBP1KD467, or scrambled shRNA (Figures 1H–1K). Several stimulation rates were tested because distinct molecular mechanisms have been proposed to mediate SV retrieval at different stimulation frequencies (Cousin, 2017; Kononenko and Haucke, 2015; Soykan et al., 2017). Although the time course of exocytosis was indistinguishable between CtBP1KD and control groups, the sypHy fluorescence decay was significantly slower in CtBP1KD neurons at all frequencies tested (Figures 1I–1K), suggesting a role for CtBP1 in SV endocytosis. Analogous experiments in cultured neurons from constitutive *Ctbp1* KO mice confirmed that conclusion (Figure S2G). Taken together, these results suggest that CtBP1 contributes to SV retrieval at a broad range of neuronal firing frequencies and is specifically required for maintaining the size of TRP during sustained neuronal activity.

Deletion of CtBP1 Induces Changes in SV Size and Distribution

Next, we performed an ultrastructural analysis of small glutamatergic spine synapses in 4–5-week-old cultured hippocampal slices obtained from *Ctbp1* KO mice and their wild-type (WT) siblings. A combination of rapid cryo-fixation, automated freeze substitution, and 3D-electron tomographic analysis was designed to accurately reveal vesicular organization at presynaptic active zones (AZs) with nanometer precision and to circumvent the introduction of morphological artifacts associated with conventional electron microscopy preparation methods requiring dehydration of the tissue at room temperature (Korogod et al., 2015; Murk et al., 2003). An analysis of gross synaptic morphology and the number of SVs in individual presynaptic glutamatergic terminals revealed no differences between *Ctbp1* KO and WT synaptic profiles (Figures 2A–2G). Electron tomographic analysis, however, revealed changes in the distribution of SVs in KO versus WT synapses (Figures 2H–2K). The KO synaptic profiles showed a significant increase in the number of membrane-proximal SVs (within 0–5, 0–40, 50–100, and 0–100 nm of the AZs; Figures 2L and 2P; Table S1). It is important to note that no statistically significant differences in the number of vesicles within 0–2 nm of the AZs were observed (Figure 2M), which is the morphological correlate of RRP. Analyses of individual SVs revealed a small, but significant, increase in the diameter of docked SVs (Figure 2O); however, no change in SV size was seen when comparing all synaptic vesicles within 0–200 nm (Table S1). Altogether, these data suggest that loss of CtBP1 does not affect the overall number of SVs in the presynaptic terminals but, rather, triggers their redistribution from membrane-distal to membrane-proximal areas. They also indicate that CtBP1 regulates the size uniformity of docked SVs.

Distinct Roles of Nuclear and Synaptic CtBP1 in Neurotransmission

Since we observed changes in the diameter of docked SVs and the size of the TRP, we next determined the effect of CtBP1 depletion on neurotransmission. We first compared the AP-evoked excitatory postsynaptic currents (EPSCs) in cultures of autaptic hippocampal neurons transduced with CtBP1KD944 shRNA or scr as a control. Unexpectedly, CtBP1KD944 neurons exhibited greater amplitudes of EPSCs compared with controls (Figure 3A). To examine whether the increase in EPSC amplitude reflected an increase in the amount of glutamate loaded into SVs or changes in postsynaptic receptors, we analyzed miniature EPSCs (mEPSCs), which represent single-fusion events. Neither the amplitudes nor the charges of mEPSCs were affected by CtBP1-depletion, indicating that the observed increase in EPSC amplitude cannot be attributed to any major changes in vesicular neurotransmitter content or postsynaptic properties (Figures 3B and 3C; Table S2). In support of the latter conclusion, quantitative, live immunolabeling of autaptic neurons with an antibody recognizing the extracellular epitope of GluAs did not uncover any significant differences in the surface expression of AMPA receptors between the groups (Figures 3E and 3F). The mEPSC frequency was not significantly altered in CtBP1944KD neurons (Figure 3D). However, the number of morphological synapses assessed as a number of co-localizing synapsin-GluA puncta in CtBP1KD944 neurons was slightly higher, suggesting increased synaptogenesis in the absence of CtBP1 (Figures 3E and 3G). The increased synapse number might contribute, at least in part, to the increase of EPSC amplitude observed in these neurons.

Next, we measured the postsynaptic current evoked by application of hypertonic sucrose, leading to the release of all docked SVs (RRP) (Rosenmund and Stevens, 1996). We detected unchanged sucrose-evoked currents (Figures 3H and 3I), which is in line with unchanged RRP measured by sypHy imaging (Figures 1E–1G) and with the unchanged number of morphologically docked SVs at CtBP1-deficient synapses (Figure 2M). The unchanged total RRP charge, but a significantly higher EPSC charge evoked by an injection of a single AP, implies increased mean vesicular release probability (Pvr; Figure 3J; Table S2). Increased Pvr is predictive of increased synaptic transmission upon isolated stimuli but leads to enhanced short-term depression upon repeated stimulation. To explore that possibility, we recorded synaptic responses induced by a 25-ms spaced pair of APs (Figure 3K). In line with the elevated Pvr, the paired pulse ratio (PPR) (i.e., the ratio of the peak amplitude of the second to the first evoked EPSC), was significantly decreased in CtBP1944KD neurons, confirming a higher degree of synaptic depression. We also analyzed the depression of neurotransmission during sustained neuronal activity by recording the EPSCs evoked by a train of 50 stimuli at 10 Hz (Figure 3L). At that frequency, only minor depression of EPSC amplitudes was evident in controls (scr), whereas a pronounced rundown of neurotransmission was measured upon depletion of CtBP1 (CtBP1KD944), which is in line with the high initial Pvr and increased PPR measured in CtBP1KD944 neurons. Thus, depletion of CtBP1 promotes synaptogenesis and elevates Pvr, resulting in increased evoked neurotransmission and contributing to the strongly enhanced short-term depression.

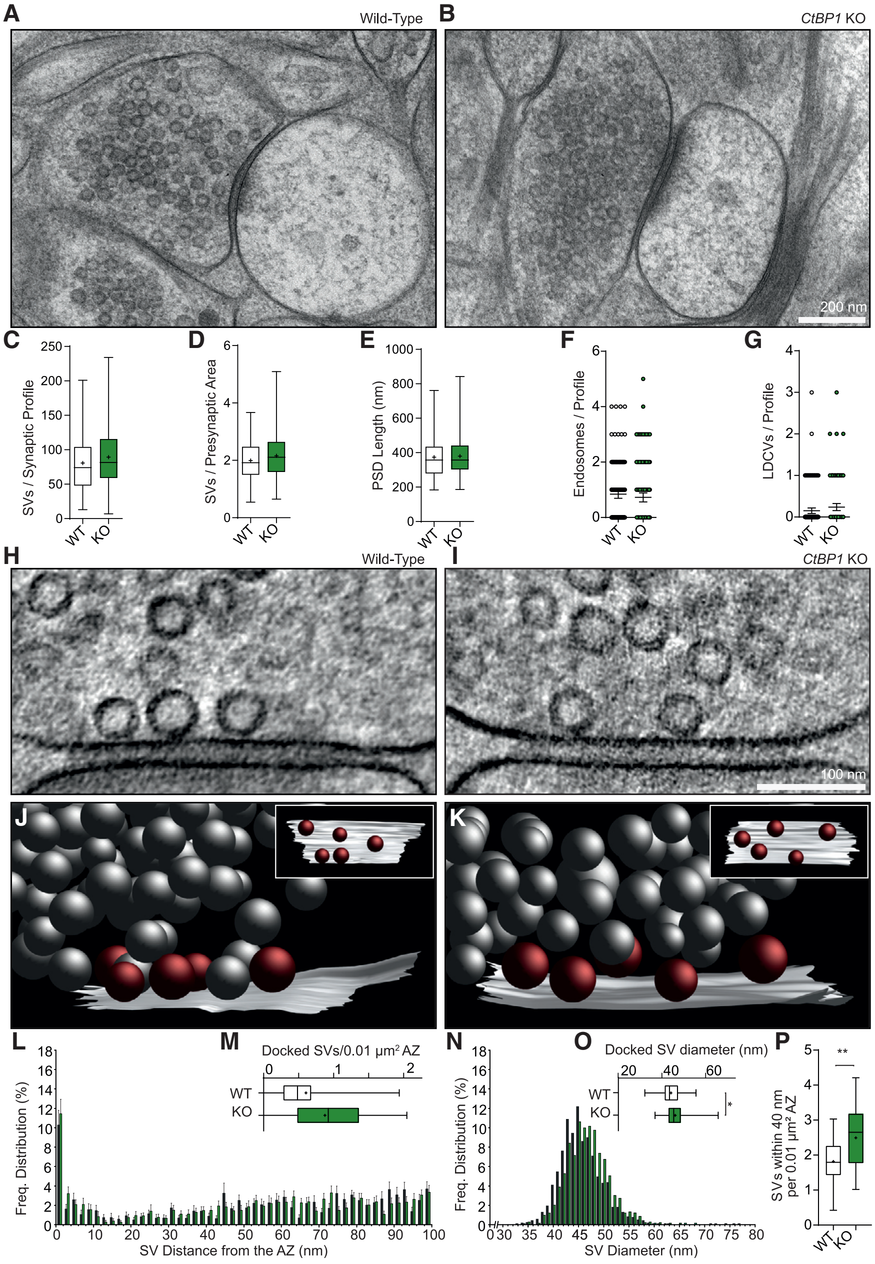

Figure 2. Ultrastructural Analysis of Synaptic Morphology and SV Distribution in Synaptic Profiles of Glutamatergic Spine Synapses in High-Pressure, Frozen, and Freeze-Substituted Hippocampal Organotypic Slice Cultures of WT and *Ctbp1* KO mice

(A and B) Electron micrographs of WT (A) and respective *Ctbp1* KO (B) synaptic profiles.

(C–G) Mean values for the number of SVs per synaptic profile (C), SV density (D), postsynaptic density (PSD) length (E), number of endosomes per synaptic profile (F), and number of large dense-core vesicles (LDCVs) per synaptic profile (G).

(H and I) Electron tomography sub-volumes of WT (H) and *Ctbp1* KO (I) synapses.

(J and K) 3D models of synaptic profiles in WT (J) and *Ctbp1* KO (K), including orthogonal views of the AZ (white), docked SVs (red), and nonattached SVs (gray).

(L–P) Graphs show spatial distribution of SVs within 100 nm of the AZ (L), mean number of docked SVs (within 0–2 nm of the AZ) per AZ area (M), frequency distribution of SV diameters within 200 nm of the AZ (N), mean diameter of docked SVs (O), and mean number SV within 0–40 nm of the AZ (P) per AZ area.

KO and WT animals were analyzed in electron micrographs of 60-nm-thick, ultrathin sections (A–G) and by 3D electron tomography (H–P). Scale bars: 200 nm in (B); 100 nm in (I).

In the plots the interquartile range and median are depicted as boxes, minimal and maximal values as whiskers and + indicates mean. In Figures 2F and 2G scatter dot plots show mean and 95% CI, and in 2 L and N bars indicate mean and SEM. Significance is indicated using asterisks: $^*p < 0.05$, $^{**}p < 0.01$.

We have previously shown that nuclear CtBP1 acts as a transcriptional corepressor and regulates the expression of plasticity-related genes that might affect synaptogenesis and neurotransmission (Ivanova et al., 2015). To discriminate between the effects of nuclear and synaptic CtBP1 on synaptic transmission, we expressed CtBP1944KD together with RNAi-resistant variants of CtBP1, which were sorted predominantly to the synapses (EGFP-CtBP1) or only to the nucleus (YFP-CtBP2(NLS)-CtBP1). In EGFP-CtBP1, the N-terminal fusion of EGFP interferes with its nuclear localization, whereas it leaves the synaptic targeting unaffected (Figure S3A) (Ivanova et al., 2015; Verger et al., 2006). The chimeric protein YFP-CtBP2(NLS)-CtBP1, which bears the nuclear localization sequence (NLS) of CtBP2, the paralog of CtBP1 in vertebrates, fused to almost full-length CtBP1, showed restricted nuclear localization (Figure S3A) (Verger et al., 2006). Although expression of synaptic EGFP-CtBP1 on a KD background led to a further increase of EPSC amplitude, expression of nuclear YFP-CtBP2(NLS)-CtBP1 fully rescued the EPSC amplitude (Figure 3A). These data indicate that the increased size of the evoked response in CtBP1KD944 neurons is a result of the depletion of the nuclear, rather than the synaptic, pool of CtBP1. Similarly, the increased number of morphological synapses as well as Pvr and PPR was substantially normalized upon expression of nuclear YFP-CtBP2(NLS)-CtBP1, indicating that depletion of nuclear CtBP1 leads to increased synaptogenesis and elevated Pvr (Figures 3G, 3J, and 3K). Expression of YFP-CtBP2(NLS)-CtBP1 also normalized the altered expression of the immediate early gene Arc and brain-derived neurotrophic factor (BDNF) in CtBP1KD944 neurons (Figures S3B and S3C), suggesting a link between CtBP1-controlled gene expression and the regulation of synaptic efficacy. We observed an increase in

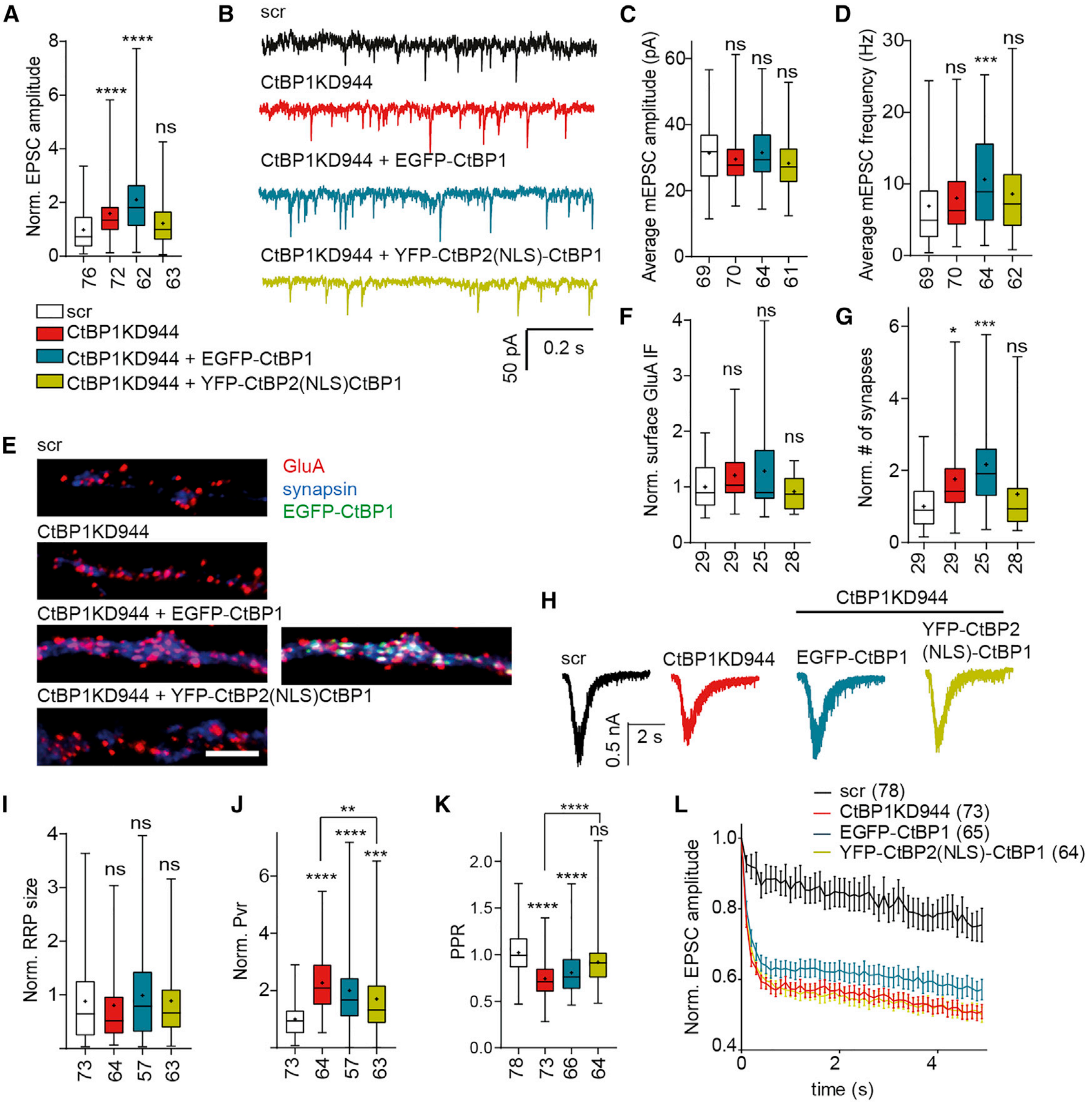

Figure 3. Synaptic and Nuclear CtBP1 Have Distinct Effects on Neurotransmission and Their Deletion Leads to Pronounced Short-Term Depression

(A) Averaged normalized evoked EPSC amplitudes from control, CtBP1KD944, EGFP-CtBP1, and YFP-CtBP2(NLS)-CtBP1 expressed in CtBP1KD944 neurons.
(B) Example traces showing spontaneous EPSCs from control, CtBP1KD944 neurons, or neurons expressing EGFP-CtBP1 and YFP-CtBP2(NLS)-CtBP1 on CtBP1KD background.
(C) Respective quantifications of average mEPSC amplitudes from the groups shown in (B).
(D) Respective quantifications of mEPSC frequency from the groups shown in (B).
(E) Autaptic neurons expressing the scr and CtBP1KD944 shRNA or the rescue variants: EGFP-CtBP1 or YFP-CtBP2(NLS)-CtBP1 on CtBP1KD944 background were live stained for surface AMPA receptors and post-fixation for synapsin to label presynapses. The overlays are shown in the indicated colors. Scale bar: 5 μm.
(F and G) Quantification of the experiment in (E). Immunofluorescence (IF) intensity of surface-expressed GluA at synapses does not differ among conditions (F), but CtBP1KD944 and expression of EGFP-CtBP1 in CtBP1KD944 neurons increase the number of synapses (G).
(H and I) Typical responses to application of 500-m OsmM sucrose for 10 s (H) and average, normalized sizes of RRP (I).
(J and K) Averaged, normalized vesicular release probability (J) and PPR (K) in control, CtBP1KD944, and EGFP-CtBP1 and YFP-CtBP2(NLS)-CtBP1 expressed in CtBP1KD944 neurons.
(L) Averaged, normalized amplitudes of EPSC evoked by a train of stimuli at 10 Hz.
In the plots the interquartile range and median are depicted as boxes, minimal and maximal values as whiskers and + indicates mean. Data points in the curve in Figures 3L are depicted as means and SEM. In the graphs comparisons with the control are indicated above each box and, comparisons between the conditions are given as horizontal bars. Significance is indicated using asterisks: nsP > 0.05, *p < 0.05, **p < 0.01, ***p < 0.001, **** p < 0.0001.

synapse number, Pvr, and PPR upon expression of synaptic EGFP-CtBP1 (Figures 3G, 3J, and 3K), which further supports the notion that not synaptic but nuclear CtBP1 controls synapse formation and/or maintenance and Pvr. The expression of EGFP-CtBP1 also led to an increase in mEPSC frequency, which might be a consequence of the concomitant strong elevation in synapse number and Pvr (Figures 3D, 3J, and 3K).

To our surprise, the expression of the nuclear construct YFP-CtBP2(NLS)-CtBP1 in CtBP1KD944 neurons that normalized the evoked neurotransmission and significantly decreased Pvr assessed upon single- or paired-pulse stimulation (Figures 3A, 3J, and 3K) did not revert the strikingly elevated depression during the train of 50 stimuli at 10 Hz (Figure 3L). In contrast, expression of synaptic EGFP-CtBP1 in CtBP1KD944, which further enhanced the evoked neurotransmission and left the increased Pvr largely unaffected, increased the steady state response to 10 Hz of stimulation by about 7% (of initial response) compared with CtBP1KD944 (Figure 3L). This is comparable with data obtained at the calyx of Held, where inhibition of endocytosis decreased the steady-state response by 10% (Hosoi et al., 2009). Taken together, the complementation experiments revealed that nuclear CtBP1 has an inhibitory effect on basal neurotransmission because of its negative effect on synapse number and SV fusion competency. Interestingly, the nuclear expression of CtBP1 (YFP-CtBP2(NLS)-CtBP1) left the enhanced depression of neurotransmission during repetitive stimulation unaffected, whereas expression of synaptic EGFP-CtBP1 ameliorated the effect of CtBP1 depletion. Because the synaptic rundown during repetitive stimulation is determined not only by the Pvr but also by the size and refill capacity of the total recycling pool of SVs, we next addressed the involvement of synaptic and nuclear CtBP1 in SV retrieval in the following imaging experiments.

Synaptic CtBP1 Is Required for Normal SV Recycling and Short-Term Plasticity of Release

To directly determine the contribution of synaptic and nuclear CtBP1 to the defect in SV retrieval observed in CtBP1KD neurons, we performed imaging experiments in neurons, where CtBP1 KD was complemented by expression of synaptic or nuclear rescue constructs. Synaptically localized EGFP-CtBP1 expressed on CtBP1KD944 background led to an ~80% restoration of Syt1 Ab uptake driven by network activity. In contrast, the expression of nuclear YFP-CtBP12(NLS)-CtBP1 failed to rescue Syt1 Ab uptake in CtBP1KD944 neurons (Figures 4A and 4B). In addition, the expression of EGFP-CtBP1 with aspartate-355-to-alanine mutation (D355A), which impairs the fission activities of CtBP1 (Bonazzi et al., 2005), also failed to restore the Syt1 Ab uptake in CtBP1KD neurons (Figures 4A and 4B), suggesting that the function of CtBP1 in fission is required for normal SV recycling. Next, we tested the ability of synaptic versus nuclear CtBP1 expression to rescue the aberrant exo-endocytosis observed upon depletion of endogenous CtBP1 (Figures 1H–1K) To that end, we used a sensor composed of synaptophysin fused to the monomeric, orange-pH-sensitive mOrange2 (sypmOr2), which we co-expressed with the EGFP- and YFP-labeled rescue constructs (Figures 4C and 4D). The fluorescence recovery after stimulation with 200 APs at 20 Hz was significantly retarded in CtBP1KD944: it did not reach full recovery during the time of imaging and had a greater recovery halftime compared with the controls (Figures 4C and 4D). The expression of synaptic EGFP-CtBP1 on the CtBP1KD944 background fully rescued the normal SV retrieval, whereas nuclear YFP-CtBP2(NLS)-CtBP1 or the fission mutant EGFP-CtBP1D355A failed to do so (Figures 4C and 4D). Altogether, these data indicate that the synaptic localization and intact fission activities of CtBP1 are crucial for its role in SV retrieval.

To re-evaluate the altered short-term plasticity measured by the electrophysiological recordings of CtBP1-depleted autaptic neurons (Figure 3L), we monitored the exocytosis of endogenous syt1 during a train of 200 APs at 10 Hz using an antibody against its luminal domain coupled to CypHer5E (Syt1 Ab-CypHer). CypHer5E is a pH-sensitive dye with maximal fluorescence at acidic pH in the vesicle lumen, and fluorescence decline upon SV exocytosis (Hua et al., 2011). Experiments were performed in the presence of bafilomycin A1 (Figure 4E) or folimycin (Figure S4) to block SV reacidification and to, thus, visualize net SV fusion. To normalize for potential differences in the initial release probability and to, thus, uncover the contribution of SV retrieval, the response amplitudes after a reference train of 40 APs at 20 Hz, which leads to the release of RRP (unchanged between control and CtBP1KD; Figures 1G, 2I, 2M, 3H, and 3I), were used for normalization, as described previously (Hua et al., 2013). This reference pulse was followed by a brief recovery period and a test stimulus of 200 APs at 10 Hz. The amplitudes of the fluorescence responses to 200 APs were strongly reduced in CtBP1KD944 compared with the control for stimuli delivered at 5, 10, or 40 Hz (Figures 4E, 4F, S4A, and S4B). The expression of YFP-CtBP2(NLS)-CtBP1 on CtBP1KD944 background did not improve that decrease, whereas the responses in KD neurons expressing EGFP-CtBP1 construct were not significantly different from the control (Figures 4E and 4F). These experiments further supported the view that synaptic CtBP1 is required for efficient SV recycling during sustained neuronal activity.

Dynamin-Dependent SV Recycling Is Unaffected in CtBP1-Deficient Neurons

The GTPase dynamin has a key role in the reformation of SVs by catalyzing the fission of SV membranes from the plasma membrane and endosomal structures (Gan and Watanabe, 2018; Kononenko and Haucke, 2015). In non-neuronal cells, CtBP1 was described as an accessory protein in the assembly of the dynamin-independent fission machinery, which includes molecules such as ADP ribosylation factor (Arf), phospholipase D (PLD), and lysophosphatidic acid acyltransferase (LPAAT) (Haga et al., 2009; Pagliuso et al., 2016; Valente et al., 2012). To investigate a possible link between CtBP1 and the established presynaptic endocytic machinery, we assessed the nanoscale localization of CtBP1 with respect to other membranous structures implicated in SV recycling. To that end, we performed super-resolution, dual-color stimulated emission depletion (STED) microscopy of neurons labeled with antibodies against CtBP1, the SV protein Syt1, and several endosome markers, which was followed by co-localization modeling. Dynamin1 labeling was used to visualize the classic endocytic machinery

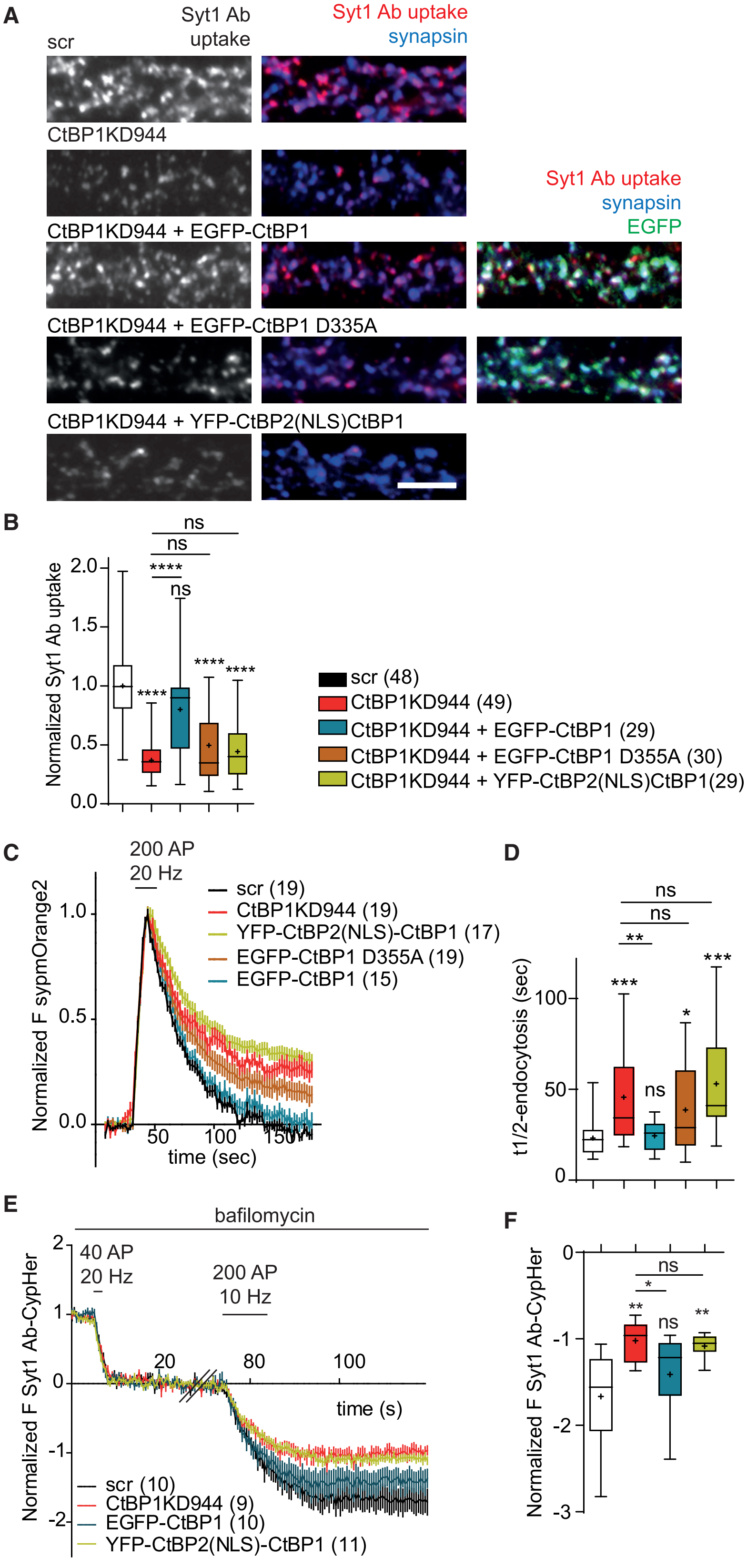

Figure 4. Synaptic CtBP1 Regulates SV Recycling

(A) Syt1 Ab uptake was used to evaluate the efficacy of SV recycling in control, CtBP1KD944, and CtBP1KD944 neurons expressing the rescue constructs: EGFP-CtBP1 and YFP-CtBP2(NLS)-CtBP1. Neurons were stained for synapsin to label synapses. Colored images represent overlays. Scale bar: 5 μm.

(B) Expression of EGFP-CtBP1 rescues the Syt1 Ab uptake in CtBP1KD944 neurons up to 80% of the control levels. The fission-deficient mutant EGFP-CtBP1D355A has a reduced rescue capacity compared with EGFP-CtBP1. Expression of the nuclear rescue, YFP-CtBP2(NLS)-CtBP1, does not compensate for the decreased Syt1 Ab uptake in CtBP1KD944.

(C) Average sypm-Orange2 responses to 200 APs at 20 Hz from control, CtBP1KD944, or CtBP1KD944 neurons expressing EGFP-CtBP1, EGFP-CtBP1D355A, or YFP-CtBP2(NLS)-CtBP1.

(D) The endocytic half times, $t_{1/2}$ from the experiment in (C) indicated that the rate of endocytosis was significantly lower in CtBP1KD944 compared with the control. Although expression of EGFP-CtBP1 in CtBP1KD944 cells rescued the endocytosis rate, expression of EGFP-CtBP1D355A or YFP-CtBP2(NLS)-CtBP1 did not.

(E) Visualization of short-term depression of exocytosis in CtBP1KD944 and upon expression of rescue constructs. Plotted are average Syt1 Ab-CypHer responses to 40 APs at 20 Hz (a reference response), followed by a 60-s rest period and 200 APs at 10 Hz in the presence of bafilomycin A1. The traces were normalized to the amplitudes of the reference responses in each condition.

(F) The absence of synaptic CtBP1 led to a reduction of the plateau fluorescence responses in experiment (E).

In the plots the interquartile range and median are depicted as boxes, minimal and maximal values as whiskers and + indicates mean. Data points in curves in Figures 4C and 4E are depicted as means and SEM In the graphs comparisons with the control are indicated above each box and, comparisons between the conditions are given as horizontal bars. Significance is indicated using asterisks: nsP > 0.05, *p < 0.05, **p < 0.01, ***p < 0.001, **** p < 0.0001.

(Figure 5A). Because many of the components of the CtBP1-associated fission machinery were shown to coordinate the endosomal trafficking of membrane proteins, we also labeled the neurons with markers for early (rab5), late (rab7), and recycling (rab22) endosomes (Figure 5A). Before staining, neuronal cultures were first silenced with APV ((2*R*)-amino-5-phosphonovaleric acid; (2*R*)-amino-5-phosphonopentanoate) and CNQX (6-cyano-7-nitroquinoxaline-2,3-dione) for 10 min to reduce the intersynaptic variability induced by the endogenous network activity. We analyzed the distance of CtBP1 to other markers at rest and also monitored the co-localization in cells fixed 30 s after stimulation with 200 APs at 40 Hz (Figure S5). Overall, CtBP1 localized in close proximity (0–200 nm) to dynamin1 and Syt1, whereas all endosome markers we probed for were much more distant (100–500 nm) (Figures 5A, 5B, and S5A–S5E). Synaptic stimulation did not affect the co-localization of CtBP1 with dynamin1 and Syt1 but led to a significant increase in the distance between CtBP1 and endosome markers rab5 and rab7, but not rab22 (Figures S5A–S5E). Thus, CtBP1 likely acts at the membrane domain marked by Syt1 and dynamin1, indicating its role in the retrieval of exocytosed SVs. Poor baseline co-localization of CtBP1 with endosomal markers rab5, rab7, and rab22 and subsequent increase of distance upon neuronal stimulation suggest a role for CtBP1 in the formation of vesicular carriers, rather than its constitutive association with intracellular membranous structures.

Given that CtBP1 was reported to regulate membrane trafficking in dynamin-independent exocytic and endocytic pathways (Bonazzi et al., 2005), the high synaptic co-localization with dynamin1 was unexpected. Therefore, to test whether CtBP1 contributes to the presynaptic dynamin-dependent endocytosis, we quantified the Syt1 Ab-CypHer uptake in control and CtBP1KD944 neurons treated with the potent dynamin inhibitor dynole 34-2 (Figures 5C and 5D). Because inhibition of dynamin increases the membrane stranding of SV proteins because of impaired retrieval (Raimondi et al., 2011), we used Syt1 Ab-CypHer uptake to determine, specifically, the fraction of Syt1 retrieved through dynamin-independent endocytosis. Dynole 34-2 had a comparable effect in control and in CtBP1KD944 neurons and reduced the Syt1 Ab-CypHer uptake by more than 80% (Figure 5D). The large effect of dynamin inhibition in both conditions confirms the principal requirement of dynamin for efficient SV retrieval at the presynapse. However, because the effects of CtBP1KD and dynole 34-2 were not completely additive, but rather cooperative, and considering the high degree of co-localization observed for CtBP1 and dynamin, we propose that, despite their involvement in independent machineries, they might act in concert at the same membrane domain to mediate effective SV retrieval.

CtBP1 Promotes Retrieval of SVs by Activation of Presynaptic PLD1

Given the established role of CtBP1 in membrane trafficking in non-neuronal cells, we hypothesized a role of CtBP1-based fission machinery in SV recycling. To test that hypothesis, we first treated control and CtBP1-depleted neurons with brefeldin A (BFA), a fungal antibiotic interfering with intracellular membrane trafficking. BFA targets several proteins involved in membrane trafficking, including CtBP1. It induces ADP-ribosylation of CtBP1 (also known as BFA-ADP-ribosylation substrate [BARS]), which interferes with the assembly of CtBP1-based fission complex and results in inhibition of vesicle formation (Colanzi et al., 2013; Spanò et al., 1999). We applied BFA (2.5 μM) only 5 min before and during the image acquisition, which we reasoned is too short a time to influence synaptic function by changes in gene expression or soma-to-synapse trafficking. Thus, the effect of BFA treatment more likely reflects an acute inhibition of CtBP1 and the associated fission machinery at the presynapse. In agreement with previous reports (Kononenko et al., 2013; Park et al., 2016) (but see Kim and Ryan, 2009 for lack of effect of BFA on vGLUT-pHluorin), BFA treatment significantly affected the post-stimulus fluorescence decay of sypHy in control neurons (Figure 6A), indicating that BFA slows down the retrieval of exocytosed SVs. In contrast, the sypHy fluorescence decay was not further affected by BFA in CtBP1KD neurons (Figure 6B), suggesting that CtBP1-based fission machinery mediates to a great extent the effect of BFA.

The precise molecular mechanism of CtBP1-mediated membrane fission is still not fully understood. It was suggested that CtBP1-based fission complex drives membrane budding and fission by catalyzing the remodeling of membrane lipids, which leads to formation of fission-prone membrane domains. In non-neuronal cells, CtBP1 was shown to interact and activate the phosphodiesterase activity of phospholipase D1 (PLD1), an enzyme catalyzing the conversion of phosphatidylcholine (PC) into the fusogenic phosphatidic acid (PA) (Donaldson, 2009; Haga et al., 2009; Raben and Barber, 2017). Although PLD1 was shown to have a role in the control of neurotransmitter release in *Aplysia* (Humeau et al., 2001) and in the secretion of neuropeptides in chromaffin cells (Zeniou-Meyer et al., 2007), its function in the regulation of SV recycling in mammalian synapses has not been investigated yet. Therefore, next, we tested the involvement of PLD1 in SV recycling and its link to CtBP1-dependent SV retrieval. Acute application of VU 0155069 (1 μM for 5 min), a specific inhibitor of PLD1, led to a 2-fold decrease in the rate of sypHy retrieval in control neurons, whereas it had no effect on the endocytosis rate in CtBP1KD neurons (Figures 6C and 6D).

Considering the activity-induced recruitment of CtBP1 to nanodomains co-labeled with dynamin1 and Syt1 and its dissociation from the endosome markers rab5 and rab7, we hypothesized that CtBP1 localizes to the membrane regions, in which endocytosis of newly released SV proteins takes place. To test whether this is indeed the case, we performed imaging with fluorescently labeled mCLING: a lipophilic reacidification-independent probe suitable for STED nanoscopy of endocytic organelles (Revelo et al., 2014). We loaded mCLING into the synapses of APV and CNQX silenced (for 10 min) controls and CtBP1KD944 neurons by stimulation with 200 APs at 40 Hz and fixed them 30 s later. The mCLING labeling was notably reduced in the synapses in CtBP1KD944 neurons in comparison to the control (Figures 6E and 6F) but was again evident upon the expression of the shRNA-resistant EGFP-CtBP1 construct on a CtBP1KD944 background (Figure 6G). We next performed dual-color STED nanoscopy, followed by co-localization modeling, to assess the co-distribution of mCLING and EGFP-CtBP1 (Figure 6G). This

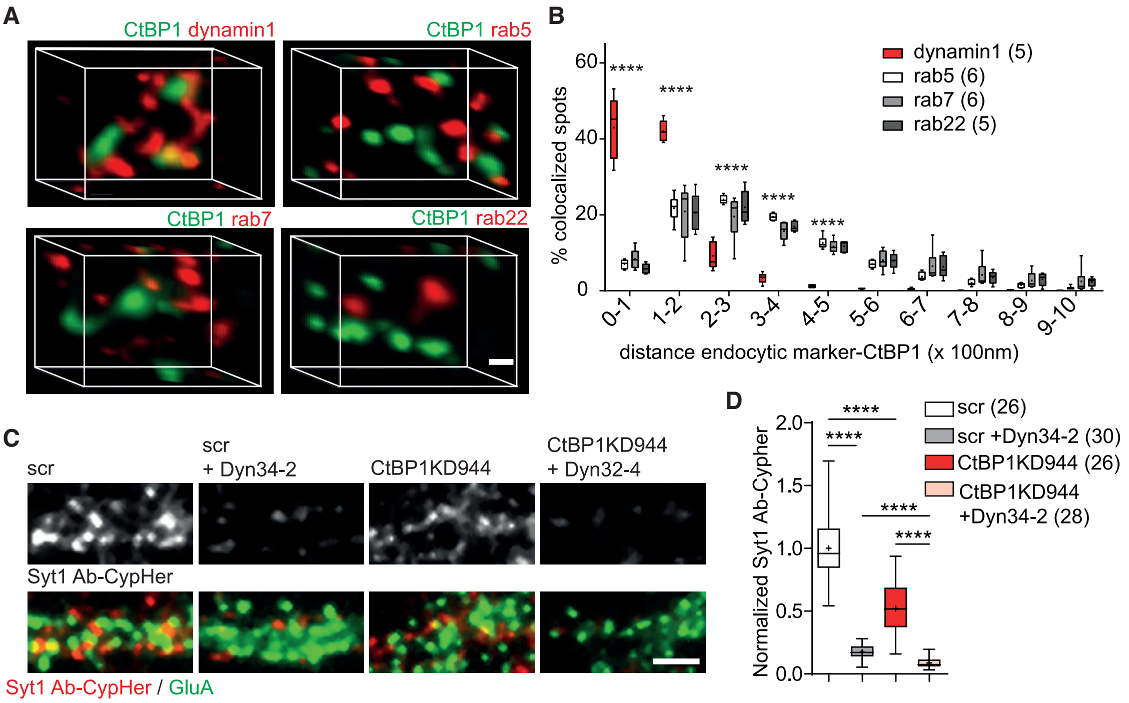

Figure 5. CtBP1 and Dynamin Act at the Same Membrane Domain in an Independent, but Likely Cooperative, Manner

(A) Orthographic views of the distribution of synaptic CtBP1 and the endocytic markers dynamin1, rab5, rab7, and rab22 in neurons stimulated with 200 APs at 40 Hz. Punctate staining was detected as "spots," and the co-localization was assessed as a distance from the CtBP1-labeled spots (synaptic distance) <1 μm.

(B) The histogram shows the distribution of synaptic puncta co-localizing with CtBP1, binned according to the distance to CtBP1. A significantly smaller distance to CtBP1 is evident for dynamin1 (0–100 and 100–200 nm distance to CtBP1) compared with the other endosome markers.

(C) Images of Syt1 Ab-CypHer uptake in control and CtBP1KD944 neurons untreated or treated with dynole 34-2 (C, 30 μM) for 1 h. Live staining for surface GluA receptors was used to mark synapses. Overlays are shown as colored images.

(D) Dynole 34-2 inhibits endocytosis in control and CtBP1KD944 neurons. The residual endocytosis is significantly lower upon Dynole 34-2 application in CtBP1944KD, suggesting an interaction of treatments.

Scale bars: 0.1 μm in (A); 5 μm in (C).

In the plots the interquartile range and median are depicted as boxes, minimal and maximal values as whiskers and + indicates mean. In the graph 5B comparisons are indicated above each group and in 5D comparisons between the conditions are given as horizontal bars. Significance is indicated using asterisks: **** $p < 0.0001$.

analysis revealed a significant negative correlation between the intensity of mCLING and the distance to individual EGFP-CtBP1 puncta, which supports a role of CtBP1 in SV endocytosis (Figure 6I).

Phosphorylation of CtBP1 at serine 147 (S147), mediated by the kinase Pak1, was found to strongly increase the capacity of CtBP1 to stimulate membrane fission by increasing its ability to activate PLD1 (Haga et al., 2009; Liberali et al., 2008). To test the importance of this regulation at the presynapse we compared the mCLING labeling in neurons expressing the RNAi resistant EGFP-CtBP1 or EGFP-CtBP1S147A construct on CtBP1KD944 background. The mCLING labeling was reduced by 80% in cells expressing EGFP-CtBP1S147A as compared to cells expressing EGFP-CtBP1 (Figures 6G and 6H) indicating a lower ability of this mutant to rescue stimulus-induced membrane retrieval upon CtBP1KD. Moreover, the co-distribution between mCLING and S147A mutant was shifted toward higher distances compared to EGFP-CtBP1 (Figure 6J), which likely reflects impaired recruitment to the sites of endocytosis. Taken together, these data indicate that the presence of CtBP1 at the endocytic sites and its phosphorylation at S147 are key factors determining the efficacy of SV retrieval.

Phosphorylation of CtBP1 Regulates Its Distribution between the CAZ and the Presynaptic Endocytic Sites

CtBP1 is recruited to the synapse via a direct interaction with the presynaptic scaffolding proteins Bsn and Pclo (Ivanova et al., 2015; tom Dieck et al., 2005). Despite the tight functional coupling between SV fusion and endocytosis, it is well established that the two processes take place at distinct membrane domains within the presynapse (Haucke et al., 2011; Maritzen and Haucke, 2018). Thus, the association of CtBP1 with Bsn and Pclo, which are established components of the SV release sites, is seemingly in disagreement with the proposed function of CtBP1 in SV endocytosis. To address that apparent ambiguity, we performed the following series of experiments. First, we performed co-immunoprecipitation (coIP) of Bsn with EGFP-CtBP1, overexpressed in primary cortical cultures in a basal state or upon a treatment with the Pak1 inhibitor IPA3 for 1 h (Figure 7A). In a basal state, a considerable coIP of CtBP1 with

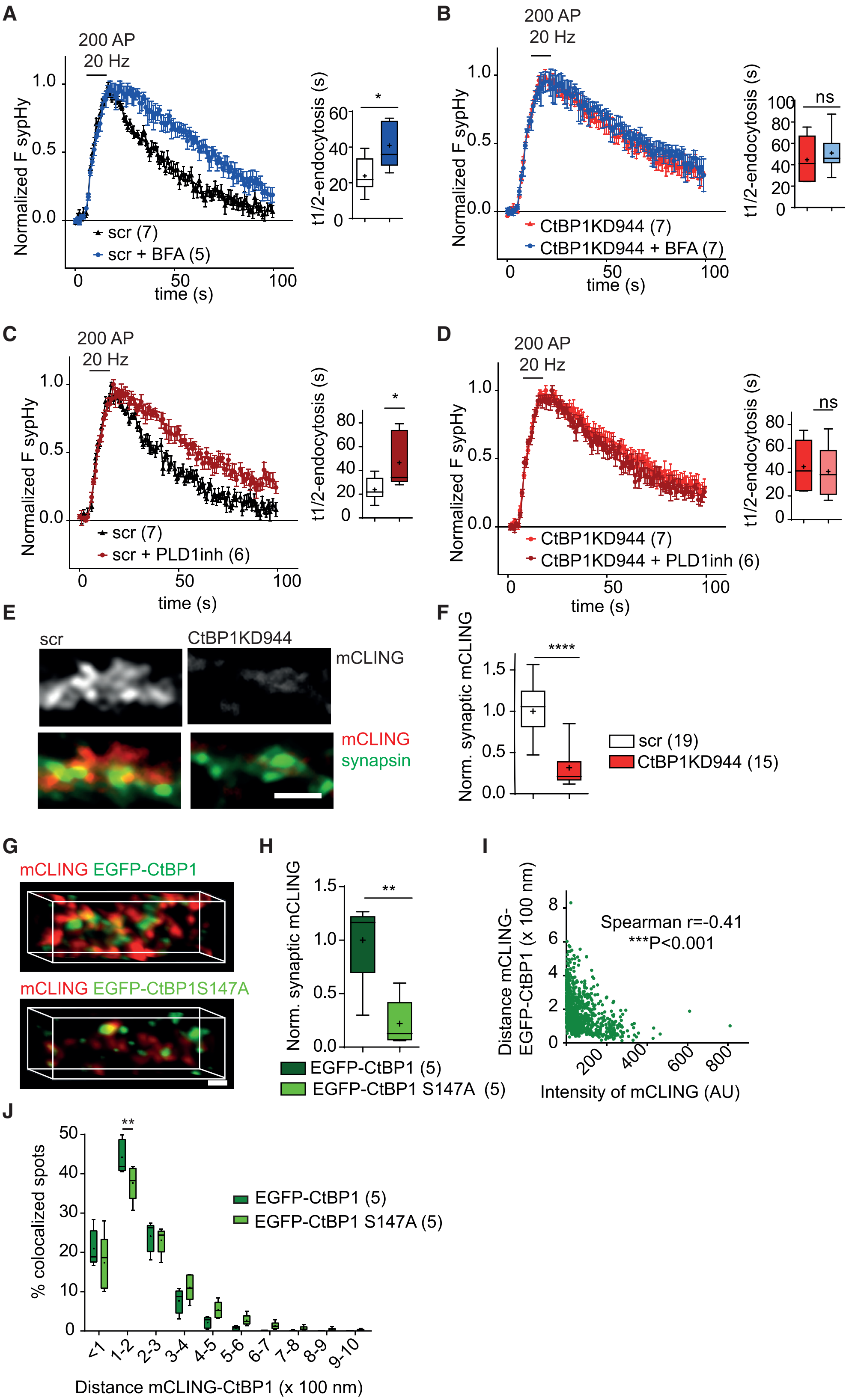

(legend on next page)

PLD1, but only a low binding to Bsn, was detected. The IPA3 treatment visibly reduced the overall serine/threonine phosphorylation of CtBP1 (Figures 7C and 7D). Consistent with the requirement for Pak1-dependent phosphorylation of CtBP1 for its association with PLD1, IPA3 reduced the coIP of PLD1 with CtBP1 to an undetectable minimum but increased the association of CtBP1 with Bsn (Figures 7A and 7B). This indicates that the phosphorylation of CtBP1 by Pak1 acts as a molecular switch, which triggers its dissociation from Bsn and binding to PLD1. To further test that hypothesis, we compared the nanoscale co-localization of EGFP-CtBP1 or S147A mutant with endogenous Bsn at synapses of acutely silenced neurons before and upon stimulation with 200 APs at 40 Hz. Consistent with our previously published observations, stimulation led to a tighter co-localization of EGFP-CtBP1 and Bsn (Figures 7E and 7F) (Ivanova et al., 2015). EGFP-CtBP1S147A showed a greater co-localization with Bsn than EGFP-CtBP1 in silenced cells, and no effect on its co-distribution with Bsn was observed upon stimulation (Figures 7E and 7F). This supports our view that Pak1-mediated phosphorylation of S147 favors a redistribution of CtBP1 from Bsn toward PLD1, thus, promoting SV retrieval through activation of PLD1.

DISCUSSION

Nuclear CtBP1 Restricts Synaptogenesis, whereas Synaptic CtBP1 Promotes SV Retrieval

In this study, we investigated the effect of CtBP1 depletion on synaptic function using KD and KO approaches. Neurons lacking CtBP1 had a normal overall morphology but showed a significant shift in the distribution of SVs toward the AZs and enlargement of the docked SVs at rest. Interestingly, a similar change in the distribution of SVs was also observed after treatment with BFA (Rampérez et al., 2017), which, as shown here, inhibits SV recycling via CtBP1 and upon depletion of Arf6, a component of the CtBP1-dependent fission machinery and an alternative activator of PLD1 (Haga et al., 2009; Tagliatti et al., 2016; Valente et al., 2012). Thus, it is tempting to speculate that insufficient PLD1 activity in the absence of CtBP1 might cause this phenotype. The efficiency of fission during vesicle budding crucially affects the size of the resulting vesicular structures. In line with that, enlarged SVs were observed in mutants of dynamin, AP180, and syndapin, which have been implicated in different steps of SV reformation, like fission, recruitment of the clathrin-coat, or induction/sensing of membrane curvature (Ferguson et al., 2007; Koch et al., 2011; Zhang et al., 1998). Thus, an involvement of CtBP1 in the fission of the SV membranes, might explain the changes in SV size observed in *Ctbp1* KO synapses.

Interference with CtBP1 expression in cultured neurons revealed its multifaceted role in the regulation of synaptogenesis and neurotransmission. A rescue strategy with CtBP1 fusion proteins selectively sorted to nucleus or synapses revealed distinct roles for CtBP1 in these spatially separated neuronal compartments. Nuclear CtBP1 restricted synaptogenesis and presynaptic vesicular release probability, possibly by repressing the expression of plasticity-related genes, such as neurotrophins or neurotransmitter receptors (Ivanova et al., 2015). In line with that, the expression of the nuclear rescue construct YFP-CtBP2(NLS)-CtBP1 could normalize the higher number of morphologically identified excitatory synapses, the enlarged amplitudes of the evoked EPSCs, and the higher Pvr and PPR observed in CtBP1KD944 neurons. Notably, the expression of the synaptic rescue (EGFP-CtBP1) on the CtBP1KD944 background tended to enhance the effect of CtBP1 depletion on synapse density and EPSC amplitude, suggesting a dominant-negative effect of this construct on the nuclear functions of CtBP1. One possible explanation of this effect is that the EGFP-CtBP1 binds to the nuclear CtBP1-interacting partners and promotes their cytoplasmic retention. However, expression of this construct on a CtBP1KD944 background compensated for the defects in SV retrieval and ameliorated the enhanced short-term depression of neurotransmission upon repetitive stimulations. This indicates a positive effect of synaptic CtBP1 on neurotransmission. Based on that, we can speculate that the recently reported activity-induced redistribution of CtBP1 from nucleus to presynapses exerts a dual-positive effect on neurotransmission (Ivanova et al., 2015). Thus, during bursts of intense neuronal activity the reduced nuclear abundance of CtBP1 will lead to a release of the transcriptional

Figure 6. CtBP1 Promotes SV Retrieval by Activation of PLD1

(A–D) Average sypHy responses to 200 APs at 20 Hz were recorded, and quantification of $t_{1/2}$ of recovery was performed upon treatment with BFA (A and B) or PLD1 inhibitor (C and D) in control (A and C) or CtBP1KD944 (B and D) neurons. SV retrieval was significantly delayed in BFA-treated neurons (A) but not further affected in BFA treated CtBP1KD944 neurons (B). Treatment with a PLD1 inhibitor affected SV retrieval in control neurons (C) but not in CtBP1KD944 neurons (D). The same controls were plotted in (A) and (C) as well as in (B) and (D), respectively.

(E) The endocytic probe mCLING-DY654 was loaded by stimulation of control and CtBP1KD944 neurons with 200 APs at 40 Hz. Synapses were stained with synapsin Ab. Synapses in CtBP1KD944 neurons show a reduction in the mCLING labeling.

(F) Quantification of synaptic mCLING IF in (E).

(G) Orthographic views of synaptic EGFP-CtBP1 or EGFP-CtBP1S147A (S147A) expressed in CtBP1KD944 neurons and the endocytic probe mCLING-ATTO647N, loaded by stimulation with 200 APs at 40 Hz.

(H) Quantification of the mCLING intensities from EGFP-CtBP1- and S147A-labeled synapses in (G).

(I) Correlation of mCLING intensities and the distances to EGFP-CtBP1. The intensity of the endocytic probe was inversely correlated with the distance to EGFP-CtBP1.

(J) The histogram shows the distribution of mCLING puncta co-localizing with EGFP-CtBP1 or S147A, binned according to the distance mCLING-CtBP1. Note the shift in the histogram of EGFP-CtBP1 toward closer distances.

Scale bars: 2 μm in (E); 0.1 μm in (G).

In the plots the interquartile range and median are depicted as boxes, minimal and maximal values as whiskers and + indicates mean. Data points in curves in Figures 6A–6D are depicted as means and SEM. Significance is indicated using asterisks: nsP > 0.05, *p < 0.05, **p < 0.01, **** p < 0.0001.

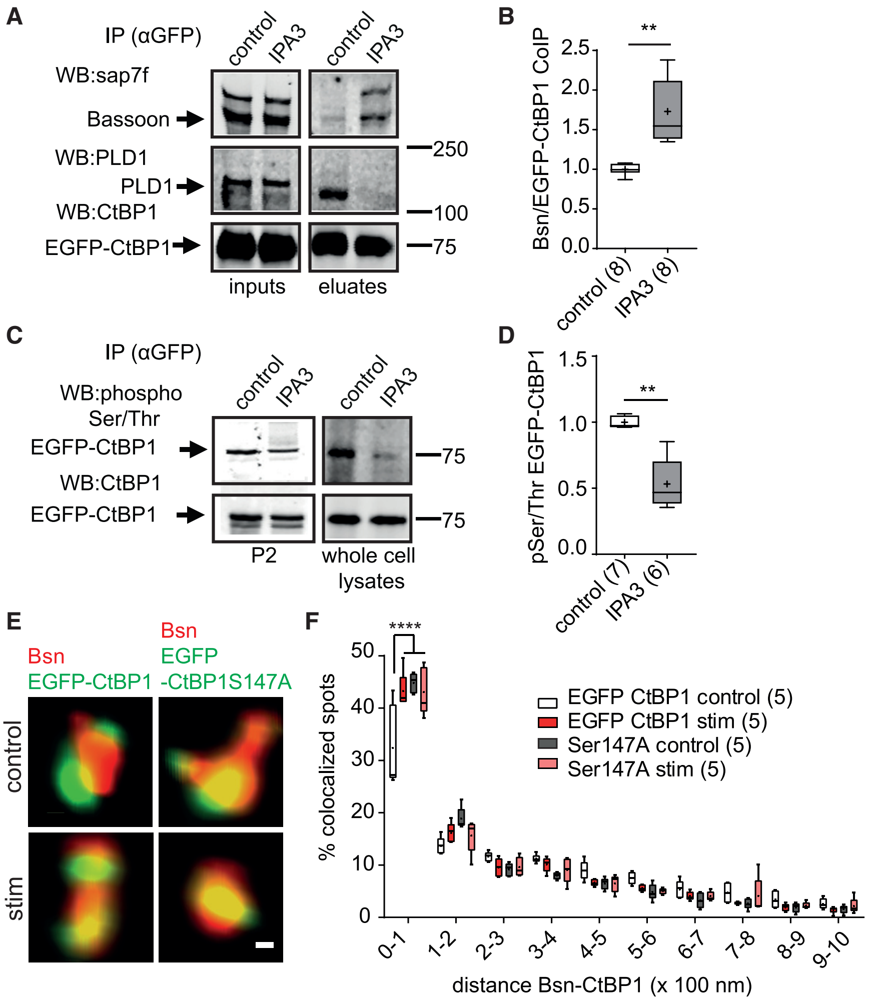

Figure 7. PAK1 Phosphorylation Mediates a Switch in the Association of CtBP1 with Bsn and PLD1

(A and B) Inhibition of Pak1 increases the binding of EGFP-CtBP1 to Bsn and reduces its binding to PLD1. (A) Co-IP with EGFP antibodies was performed from neuronal cultures expressing EGFP-CtBP1 and treated or not with the Pak1 inhibitor IPA3 (50 μM, 1 h). (B) Quantification of the binding of Bsn to CtBP1.

(C and D) IP with EGFP antibodies was performed from whole cell lysates or P2 fractions of neuronal cultures expressing EGFP-CtBP1 and treated or not with the Pak1 inhibitor IPA3 (50 μM for 1 h). (C) The western blots were probed with a pan anti Ser/Thr Ab to visualize the phospho-Ser/Thr levels of CtBP1. (D) Quantification of the Ser/Thr phosphorylation of CtBP1 is shown.

(E) The two-color STED images show a tighter co-localization of EGFP-CtBP1 with Bsn after stimulation with 200 APs at 40 Hz compared with cells at rest. EGFP-CtBP1S147A displays a tight co-localization with Bsn independently of neuronal activity.

(F) The histogram shows the relative distribution of Bsn puncta co-localizing with EGFP-CtBP1 or S147A at rest and upon stimulation.

Scale bar: 40 nm. In the plots the interquartile range and median are depicted as boxes, minimal and maximal values as whiskers, and + indicates mean. Significance is indicated by asterisks: $**p < 0.01$ and $****p < 0.0001$.

block of neuroplasticity-related genes, whereas the enhanced synaptic targeting will facilitate SV recycling.

CtBP1-Mediated Membrane Fission and PLD1 Activation Are Required for SV Retrieval

Our data indicate that CtBP1-mediated membrane fission and activation of PLD1 have an important contribution to the effective SV retrieval at the presynapse. We provide the following evidence supporting that view: (1) the CtBP1D355A fission-deficient mutant failed to rescue SV retrieval in CtBP1KD944, (2) the CtBP1S147A mutant that could not recruit PI4KIIIβ/ARF6 and activate PLD1 failed to rescue endocytosis visualized with mCLING, and (3) the pharmacological inhibition of CtBP1-based fission complex, using BFA or inhibition of PLD1 activity, phenocopied the aberrant SV retrieval observed in CtBP1KD. Our data also indicate a role for PLD1 in SV recycling at hippocampal synapses. PLD1 was detected in synaptic plasma membranes isolated from rat synaptosomes, and interference with PLD1 was shown to affect acetylcholine release from nerve ganglia in *Aplysia* (Humeau et al., 2001). However, PLD1 was mainly discussed in the context of exocytosis in neurons and chromaffin cells (Zeniou-Meyer et al., 2007). Our data indicate a role for PLD1 in SV retrieval in hippocampal synapses and reveal a requirement for CtBP1-mediated activation of PLD1 in that process. The activation of PLD1 depends on Pak1-mediated phosphorylation of CtBP1. It is unclear whether and how Pak1 activity is regulated at the presynapse, but based on our findings, we can speculate that the level of presynaptic Pak1 activity could regulate the SV retrieval and thereby modulate short-term plasticity of neurotransmission. Interestingly, the phosphorylation of S147 of CtBP1 by Pak1, which is necessary for PLD1 activation, also induces dissociation of CtBP1 from Bsn, which anchors it to the active zones. This suggests that Pak1 activity might induce rapid activation of PLD1 near presynaptic release sites and thereby link SV fusion and retrieval in time, space, and extent.

CtBP1-Mediated Lipid Reorganization in SV Retrieval

CtBP1-based fission machinery was proposed to act in a dynamin-independent manner at the Golgi and plasma membrane in non-neuronal cells (Bonazzi et al., 2005; Haga et al., 2009; Yang et al., 2008). However, the fluid-phase endocytosis switched from a CtBP1-dependent to a dynamin-dependent mechanism in fibroblasts in which CtBP1 was knocked out (Bonazzi et al., 2005), suggesting a tight interaction between those pathways. Thus, it is possible that CtBP1- and dynamin-based fission machineries converge in their action at the presynapse, where particularly potent endocytosis is required for sustained SV

replenishment. CtBP1 was suggested to mediate fission of target membranes by activation of lipid enzymes, such as PLD1 and LPAAT, which generate curvature-inducing lipid modifications (Haga et al., 2009; Liberali et al., 2008; Pagliuso et al., 2016), and by their recruitment to the machinery, initiate vesicular budding and tubulation (Valente et al., 2012). PLD1 and LPAAT catalyze the production of the fusogenic PA, which, because of its conical shape, promotes the negative membrane curvature necessary for vesicle fusion and fission (Kooijman et al., 2003). In addition to its structural role, PA was also linked to the generation of $PI(4,5)P_2$, the phospholipid involved in the recruitment of numerous proteins involved in endocytosis, including dynamin (Puchkov and Haucke, 2013). Specifically, PA activates PI kinases necessary for $PI(4,5)P_2$ production (Jenkins et al., 1994; Moritz et al., 1992), and intriguingly, one of them, PI4KIIIβ, is a component of the CtBP1-based fission complex in non-neuronal cells (Valente et al., 2012). Thus, it is likely that CtBP1 promotes SV retrieval by recruitment and activation of multiple lipid-modifying enzymes, which drive the formation of a lipid-environment permissive for compensatory endocytosis. The tight co-localization of CtBP1 and dynamin and the cooperative effect of the interference with their functions on SV recycling support that view. However, future studies will be needed to gain more insight into the mechanisms linking and regulating the different fission machineries involved in SV recycling.

STAR★METHODS

Detailed methods are provided in the online version of this paper and include the following:

- KEY RESOURCES TABLE
- LEAD CONTACT AND MATERIALS AVAILABILITY
- EXPERIMENTAL MODEL AND SUBJECT DETAILS
  - Animals
  - Lentiviral particle production
  - Primary cultures and treatments
- METHOD DETAILS
  - Antibodies
  - DNA constructs
  - Ultrastructural analysis
  - Quantitative real-time PCR
  - Biochemical experimental work
  - Microscopy and image analysis
  - pHluorin imaging and analysis
  - Electrophysiology
- QUANTIFICATION AND STATISTICAL ANALYSIS
- DATA AND CODE AVAILABILITY

SUPPLEMENTAL INFORMATION

Supplemental Information can be found online at https://doi.org/10.1016/j.celrep.2020.01.079.

ACKNOWLEDGMENTS

The YFP-CtBP2(NLS)-CtBP1 construct was kindly provided by M. Crossley, University of Sydney, Australia. We thank Anika Dirks for help with maintenance of the *Ctbp1* KO mouse colony; Christiana Kontaxi for help with animal handling; Maria Jose for help with imaging; Oliver Kobler, Torsten Stoeter, and SL ELMI for providing expertise in STED imaging and tools for analysis; and Janina Juhle, Bettina Kracht, Anita Heine, and Isabel Herbert for excellent technical assistance. We also thank Renato Frischknecht, all members of the Presynaptic plasticity group, and the Department Neurochemistry and Molecular Biology at LIN for useful discussions. This research was supported by the German Research Council (grant GRK2162, SFB 779/A06, FE1335/1, and FE1335/3 to A.F. and SFB958 to C.R.), Wellcome Trust (grant 204954/Z/16/Z to M.A.C.), and Leibniz SAW (grants to A.F. and E.D.G.).

AUTHOR CONTRIBUTIONS

Conceptualization, D.I. and A.F.; Methodology, D.I., C.I., M.C., C.M.-V., D.G., M.A.C., B.C., and A.F.; Investigation, D.I., C.I., M.C., A.R., D.G., and B.C.; Writing – Original Draft, D.I. and A.F.; Writing – Review & Editing, all authors; Funding Acquisition, M.A.C., C.R., E.D.G., and A.F.

DECLARATION OF INTEREST

The authors declare no competing interests.

Received: November 6, 2018
Revised: December 12, 2019
Accepted: January 22, 2020
Published: February 18, 2020

REFERENCES

Antonny, B., Burd, C., De Camilli, P., Chen, E., Daumke, O., Faelber, K., Ford, M., Frolov, V.A., Frost, A., Hinshaw, J.E., et al. (2016). Membrane fission by dynamin: what we know and what we need to know. EMBO J. *35*, 2270–2284.

Bekkers, J.M., and Stevens, C.F. (1991). Excitatory and inhibitory autaptic currents in isolated hippocampal neurons maintained in cell culture. Proc. Natl. Acad. Sci. USA *88*, 7834–7838.

Bonazzi, M., Spanò, S., Turacchio, G., Cericola, C., Valente, C., Colanzi, A., Kweon, H.S., Hsu, V.W., Polishchuck, E.V., Polishchuck, R.S., et al. (2005). CtBP3/BARS drives membrane fission in dynamin-independent transport pathways. Nat. Cell Biol. *7*, 570–580.

Burrone, J., Li, Z., and Murthy, V.N. (2006). Studying vesicle cycling in presynaptic terminals using the genetically encoded probe synaptopHluorin. Nat. Protoc. *1*, 2970–2978.

Chinnadurai, G. (2009). The transcriptional corepressor CtBP: a foe of multiple tumor suppressors. Cancer Res. *69*, 731–734.

Colanzi, A., Grimaldi, G., Catara, G., Valente, C., Cericola, C., Liberali, P., Ronci, M., Lalioti, V.S., Bruno, A., Beccari, A.R., et al. (2013). Molecular mechanism and functional role of brefeldin A-mediated ADP-ribosylation of CtBP1/BARS. Proc. Natl. Acad. Sci. USA *110*, 9794–9799.

Cousin, M.A. (2017). Integration of synaptic vesicle cargo retrieval with endocytosis at central nerve terminals. Front. Cell. Neurosci. *11*, 234.

Dick, O., Hack, I., Altrock, W.D., Garner, C.C., Gundelfinger, E.D., and Brandstätter, J.H. (2001). Localization of the presynaptic cytomatrix protein Piccolo at ribbon and conventional synapses in the rat retina: comparison with Bassoon. J. Comp. Neurol. *439*, 224–234.

Donaldson, J.G. (2009). Phospholipase D in endocytosis and endosomal recycling pathways. Biochim. Biophys. Acta *1791*, 845–849.

Egashira, Y., Takase, M., and Takamori, S. (2015). Monitoring of vacuolar-type H+ ATPase-mediated proton influx into synaptic vesicles. J. Neurosci. *35*, 3701–3710.

Fejtova, A., Davydova, D., Bischof, F., Lazarevic, V., Altrock, W.D., Romorini, S., Schöne, C., Zuschratter, W., Kreutz, M.R., Garner, C.C., et al. (2009). Dynein light chain regulates axonal trafficking and synaptic levels of Bassoon. J. Cell Biol. *185*, 341–355.

Ferguson, S.M., Brasnjo, G., Hayashi, M., Wölfel, M., Collesi, C., Giovedi, S., Raimondi, A., Gong, L.W., Ariel, P., Paradise, S., et al. (2007). A selective

activity-dependent requirement for dynamin 1 in synaptic vesicle endocytosis. Science *316*, 570–574.

Gan, Q., and Watanabe, S. (2018). Synaptic vesicle endocytosis in different model systems. Front. Cell. Neurosci. *12*, 171.

Garriga-Canut, M., Schoenike, B., Qazi, R., Bergendahl, K., Daley, T.J., Pfender, R.M., Morrison, J.F., Ockuly, J., Stafstrom, C., Sutula, T., and Roopra, A. (2006). 2-Deoxy-D-glucose reduces epilepsy progression by NRSF-CtBP-dependent metabolic regulation of chromatin structure. Nat. Neurosci. *9*, 1382–1387.

Granseth, B., Odermatt, B., Royle, S.J., and Lagnado, L. (2006). Clathrin-mediated endocytosis is the dominant mechanism of vesicle retrieval at hippocampal synapses. Neuron *51*, 773–786.

Haga, Y., Miwa, N., Jahangeer, S., Okada, T., and Nakamura, S. (2009). CtBP1/BARS is an activator of phospholipase D1 necessary for agonist-induced macropinocytosis. EMBO J. *28*, 1197–1207.

Haucke, V., Neher, E., and Sigrist, S.J. (2011). Protein scaffolds in the coupling of synaptic exocytosis and endocytosis. Nat. Rev. Neurosci. *12*, 127–138.

Hildebrand, J.D., and Soriano, P. (2002). Overlapping and unique roles for C-terminal binding protein 1 (CtBP1) and CtBP2 during mouse development. Mol. Cell. Biol. *22*, 5296–5307.

Hosoi, N., Holt, M., and Sakaba, T. (2009). Calcium dependence of exo- and endocytotic coupling at a glutamatergic synapse. Neuron *63*, 216–229.

Hua, Y., Sinha, R., Thiel, C.S., Schmidt, R., Hüve, J., Martens, H., Hell, S.W., Egner, A., and Klingauf, J. (2011). A readily retrievable pool of synaptic vesicles. Nat. Neurosci. *14*, 833–839.

Hua, Y., Woehler, A., Kahms, M., Haucke, V., Neher, E., and Klingauf, J. (2013). Blocking endocytosis enhances short-term synaptic depression under conditions of normal availability of vesicles. Neuron *80*, 343–349.

Hübler, D., Rankovic, M., Richter, K., Lazarevic, V., Altrock, W.D., Fischer, K.D., Gundelfinger, E.D., and Fejtova, A. (2012). Differential spatial expression and subcellular localization of CtBP family members in rodent brain. PLoS ONE *7*, e39710.

Humeau, Y., Vitale, N., Chasserot-Golaz, S., Dupont, J.L., Du, G., Frohman, M.A., Bader, M.F., and Poulain, B. (2001). A role for phospholipase D1 in neurotransmitter release. Proc. Natl. Acad. Sci. USA *98*, 15300–15305.

Imig, C., and Cooper, B.H. (2017). 3D analysis of synaptic ultrastructure in organotypic hippocampal slice culture by high-pressure freezing and electron tomography. Methods Mol. Biol. *1538*, 215–231.

Imig, C., Min, S.W., Krinner, S., Arancillo, M., Rosenmund, C., Südhof, T.C., Rhee, J., Brose, N., and Cooper, B.H. (2014). The morphological and molecular nature of synaptic vesicle priming at presynaptic active zones. Neuron *84*, 416–431.

Ivanova, D., Dirks, A., Montenegro-Venegas, C., Schöne, C., Altrock, W.D., Marini, C., Frischknecht, R., Schanze, D., Zenker, M., Gundelfinger, E.D., and Fejtova, A. (2015). Synaptic activity controls localization and function of CtBP1 via binding to Bassoon and Piccolo. EMBO J. *34*, 1056–1077.

Ivanova, D., Dirks, A., and Fejtova, A. (2016). Bassoon and piccolo regulate ubiquitination and link presynaptic molecular dynamics with activity-regulated gene expression. J. Physiol. *594*, 5441–5448.

Jenkins, G.H., Fisette, P.L., and Anderson, R.A. (1994). Type I phosphatidylinositol 4-phosphate 5-kinase isoforms are specifically stimulated by phosphatidic acid. J. Biol. Chem. *269*, 11547–11554.

Kim, S.H., and Ryan, T.A. (2009). Synaptic vesicle recycling at CNS snapses without AP-2. J. Neurosci. *29*, 3865–3874.

Koch, D., Spiwoks-Becker, I., Sabanov, V., Sinning, A., Dugladze, T., Stellmacher, A., Ahuja, R., Grimm, J., Schüler, S., Müller, A., et al. (2011). Proper synaptic vesicle formation and neuronal network activity critically rely on syndapin I. EMBO J. *30*, 4955–4969.

Kononenko, N.L., and Haucke, V. (2015). Molecular mechanisms of presynaptic membrane retrieval and synaptic vesicle reformation. Neuron *85*, 484–496.

Kononenko, N.L., Diril, M.K., Puchkov, D., Kintscher, M., Koo, S.J., Pfuhl, G., Winter, Y., Wienisch, M., Klingauf, J., Breustedt, J., et al. (2013). Compromised fidelity of endocytic synaptic vesicle protein sorting in the absence of stonin 2. Proc. Natl. Acad. Sci. USA *110*, E526–E535.

Kooijman, E.E., Chupin, V., de Kruijff, B., and Burger, K.N. (2003). Modulation of membrane curvature by phosphatidic acid and lysophosphatidic acid. Traffic *4*, 162–174.

Korogod, N., Petersen, C.C., and Knott, G.W. (2015). Ultrastructural analysis of adult mouse neocortex comparing aldehyde perfusion with cryo fixation. eLife *4*.

Kraszewski, K., Mundigl, O., Daniell, L., Verderio, C., Matteoli, M., and De Camilli, P. (1995). Synaptic vesicle dynamics in living cultured hippocampal neurons visualized with CY3-conjugated antibodies directed against the lumenal domain of synaptotagmin. J. Neurosci. *15*, 4328–4342.

Kremer, J.R., Mastronarde, D.N., and McIntosh, J.R. (1996). Computer visualization of three-dimensional image data using IMOD. J. Struct. Biol. *116*, 71–76.

Lazarevic, V., Schöne, C., Heine, M., Gundelfinger, E.D., and Fejtova, A. (2011). Extensive remodeling of the presynaptic cytomatrix upon homeostatic adaptation to network activity silencing. J. Neurosci. *31*, 10189–10200.

Lazarevic, V., Fieńko, S., Andres-Alonso, M., Anni, D., Ivanova, D., Montenegro-Venegas, C., Gundelfinger, E.D., Cousin, M.A., and Fejtova, A. (2017). Physiological concentrations of amyloid beta regulate recycling of synaptic vesicles via Alpha7 acetylcholine receptor and CDK5/calcineurin signaling. Front. Mol. Neurosci. *10*, 221.

Leal-Ortiz, S., Waites, C.L., Terry-Lorenzo, R., Zamorano, P., Gundelfinger, E.D., and Garner, C.C. (2008). Piccolo modulation of synapsin1a dynamics regulates synaptic vesicle exocytosis. J. Cell Biol. *181*, 831–846.

Liberali, P., Kakkonen, E., Turacchio, G., Valente, C., Spaar, A., Perinetti, G., Böckmann, R.A., Corda, D., Colanzi, A., Marjomaki, V., and Luini, A. (2008). The closure of Pak1-dependent macropinosomes requires the phosphorylation of CtBP1/BARS. EMBO J. *27*, 970–981.

Maritzen, T., and Haucke, V. (2018). Coupling of exocytosis and endocytosis at the presynaptic active zone. Neurosci. Res. *127*, 45–52.

Mastronarde, D.N. (2005). Automated electron microscope tomography using robust prediction of specimen movements. J. Struct. Biol. *152*, 36–51.

Moritz, A., De Graan, P.N., Gispen, W.H., and Wirtz, K.W. (1992). Phosphatidic acid is a specific activator of phosphatidylinositol-4-phosphate kinase. J. Biol. Chem. *267*, 7207–7210.

Murk, J.L., Posthuma, G., Koster, A.J., Geuze, H.J., Verkleij, A.J., Kleijmeer, M.J., et al. (2003). Influence of aldehyde fixation on the morphology of endosomes and lysosomes: quantitative analysis and electron tomography. J. Microscopy *212*, 81–90.

Pagliuso, A., Valente, C., Giordano, L.L., Filograna, A., Li, G., Circolo, D., Turacchio, G., Marzullo, V.M., Mandrich, L., Zhukovsky, M.A., et al. (2016). Golgi membrane fission requires the CtBP1-S/BARS-induced activation of lysophosphatidic acid acyltransferase δ. Nat. Commun. *7*, 12148.

Park, J., Cho, O.Y., Kim, J.A., and Chang, S. (2016). Endosome-mediated endocytic mechanism replenishes the majority of synaptic vesicles at mature CNS synapses in an activity-dependent manner. Sci. Rep. *6*, 31807.

Puchkov, D., and Haucke, V. (2013). Greasing the synaptic vesicle cycle by membrane lipids. Trends Cell Biol. *23*, 493–503.

Pyott, S.J., and Rosenmund, C. (2002). The effects of temperature on vesicular supply and release in autaptic cultures of rat and mouse hippocampal neurons. J. Physiol. *539*, 523–535.

Raben, D.M., and Barber, C.N. (2017). Phosphatidic acid and neurotransmission. Adv. Biol. Regul. *63*, 15–21.

Raimondi, A., Ferguson, S.M., Lou, X., Armbruster, M., Paradise, S., Giovedi, S., Messa, M., Kono, N., Takasaki, J., Cappello, V., et al. (2011). Overlapping role of dynamin isoforms in synaptic vesicle endocytosis. Neuron *70*, 1100–1114.

Rampérez, A., Sánchez-Prieto, J., and Torres, M. (2017). Brefeldin A sensitive mechanisms contribute to endocytotic membrane retrieval and vesicle recycling in cerebellar granule cells. J. Neurochem. *141*, 662–675.

Renard, H.F., Johannes, L., and Morsomme, P. (2018). Increasing diversity of biological membrane fission mechanisms. Trends Cell Biol. *28*, 274–286.

Revelo, N.H., Kamin, D., Truckenbrodt, S., Wong, A.B., Reuter-Jessen, K., Reisinger, E., Moser, T., and Rizzoli, S.O. (2014). A new probe for super-resolution imaging of membranes elucidates trafficking pathways. J. Cell Biol. *205*, 591–606.

Rose, T., Schoenenberger, P., Jezek, K., and Oertner, T.G. (2013). Developmental refinement of vesicle cycling at Schaffer collateral synapses. Neuron *77*, 1109–1121.

Rosenmund, C., and Stevens, C.F. (1996). Definition of the readily releasable pool of vesicles at hippocampal synapses. Neuron *16*, 1197–1207.

Soykan, T., Kaempf, N., Sakaba, T., Vollweiter, D., Goerdeler, F., Puchkov, D., Kononenko, N.L., and Haucke, V. (2017). Synaptic vesicle endocytosis occurs on multiple timescales and is mediated by formin-dependent actin assembly. Neuron *93*, 854–866.e854.

Spanò, S., Silletta, M.G., Colanzi, A., Alberti, S., Fiucci, G., Valente, C., Fusella, A., Salmona, M., Mironov, A., Luini, A., and Corda, D. (1999). Molecular cloning and functional characterization of brefeldin A-ADP-ribosylated substrate. A novel protein involved in the maintenance of the Golgi structure. J. Biol. Chem. *274*, 17705–17710.

Stewart, S.A., Dykxhoorn, D.M., Palliser, D., Mizuno, H., Yu, E.Y., An, D.S., Sabatini, D.M., Chen, I.S., Hahn, W.C., Sharp, P.A., Weinberg, R.A., and Novina, C.D. (2003). Lentivirus-delivered stable gene silencing by RNAi in primary cells. RNA *9*, 493–501.

Tagliatti, E., Fadda, M., Falace, A., Benfenati, F., and Fassio, A. (2016). Arf6 regulates the cycling and the readily releasable pool of synaptic vesicles at hippocampal synapse. eLife *5*, e10116.

tom Dieck, S., Altrock, W.D., Kessels, M.M., Qualmann, B., Regus, H., Brauner, D., Fejtová, A., Bracko, O., Gundelfinger, E.D., and Brandstätter, J.H. (2005). Molecular dissection of the photoreceptor ribbon synapse: physical interaction of Bassoon and RIBEYE is essential for the assembly of the ribbon complex. J. Cell Biol. *168*, 825–836.

Tsuriel, S., Geva, R., Zamorano, P., Dresbach, T., Boeckers, T., Gundelfinger, E.D., Garner, C.C., and Ziv, N.E. (2006). Local sharing as a predominant determinant of synaptic matrix molecular dynamics. PLoS Biol. *4*, e271.

Valente, C., Turacchio, G., Mariggiò, S., Pagliuso, A., Gaibisso, R., Di Tullio, G., Santoro, M., Formiggini, F., Spanò, S., Piccini, D., et al. (2012). A 14-3-3γ dimer-based scaffold bridges CtBP1-S/BARS to PI(4)KIIIβ to regulate post-Golgi carrier formation. Nat. Cell Biol. *14*, 343–354.

Valente, C., Luini, A., and Corda, D. (2013). Components of the CtBP1/BARS-dependent fission machinery. Histochem. Cell Biol. *140*, 407–421.

Verger, A., Quinlan, K.G., Crofts, L.A., Spanò, S., Corda, D., Kable, E.P., Braet, F., and Crossley, M. (2006). Mechanisms directing the nuclear localization of the CtBP family proteins. Mol. Cell. Biol. *26*, 4882–4894.

Wu, Y., O'Toole, E.T., Girard, M., Ritter, B., Messa, M., Liu, X., McPherson, P.S., Ferguson, S.M., and De Camilli, P. (2014). A dynamin 1-, dynamin 3- and clathrin-independent pathway of synaptic vesicle recycling mediated by bulk endocytosis. eLife *3*, e01621.

Wu, X.S., Lee, S.H., Sheng, J., Zhang, Z., Zhao, W.D., Wang, D., Jin, Y., Charnay, P., Ervasti, J.M., and Wu, L.G. (2016). Actin is crucial for all kinetically distinguishable forms of endocytosis at synapses. Neuron *92*, 1020–1035.

Yang, J.S., Gad, H., Lee, S.Y., Mironov, A., Zhang, L., Beznoussenko, G.V., Valente, C., Turacchio, G., Bonsra, A.N., Du, G., et al. (2008). A role for phosphatidic acid in COPI vesicle fission yields insights into Golgi maintenance. Nat. Cell Biol. *10*, 1146–1153.

Zeniou-Meyer, M., Zabari, N., Ashery, U., Chasserot-Golaz, S., Haeberlé, A.M., Demais, V., Bailly, Y., Gottfried, I., Nakanishi, H., Neiman, A.M., et al. (2007). Phospholipase D1 production of phosphatidic acid at the plasma membrane promotes exocytosis of large dense-core granules at a late stage. J. Biol. Chem. *282*, 21746–21757.

Zhang, B., Koh, Y.H., Beckstead, R.B., Budnik, V., Ganetzky, B., and Bellen, H.J. (1998). Synaptic vesicle size and number are regulated by a clathrin adaptor protein required for endocytosis. Neuron *21*, 1465–1475.

STAR★METHODS

KEY RESOURCES TABLE

REAGENT or RESOURCE	SOURCE	IDENTIFIER
Antibodies		
Mouse anti-CtBP1	BD Biosciences	Cat#612042; RRID:AB_399429
Mouse anti-CtBP2	BD Biosciences	Cat#612044; RRID:AB_399431
Mouse anti-synaptotagmin1 lumenal domain Oyster550	Synaptic Systems	Cat#105311; RRID:AB_993036
Mouse anti-synaptotagmin1 lumenal domain CypHer5E-labeled	Synaptic Systems	Cat#105311CpH; RRID:AB_2199307
Mouse anti-rab5	Synaptic Systems	Cat#108011; RRID:AB_887773
Mouse anti-rab7	Abcam	Cat#ab50533; RRID:AB_882241
Mouse anti-phosphoserine/threonine	BD Biosciences	Cat#612548; RRID:AB_399843
Mouse anti-GluA Oyster 550-labeled	Synaptic Systems	Cat#182411C3; RRID:AB_2619877
Mouse anti-α-tubulin	Sigma Aldrich	Cat# T9026; RRID:N/A
Rabbit anti-CtBP1	Synaptic Systems	Cat#222002; RRID:AB_2086638
Rabbit anti-GFP	Abcam	Cat#ab6556; RRID:AB_305564
Rabbit anti-SV2B	Synaptic Systems	Cat#119103; RRID:AB_2725759
Rabbit anti-GAPDH	Abcam	Cat#ab37168; RRID:AB_732652
Rabbit anti-synaptotagmin1 lumenal domain Oyster 550-labeled	Synaptic Systems	Cat#105103C3; RRID:AB_887829
Rabbit anti-synaptotagmin 1 lumenal domain	Synaptic Systems	Cat#105102; RRID:AB_887835
Rabbit anti-dynamin1	Abcam	Cat#ab3456; RRID:AB_303818
Rabbit anti-rab22a	Abcam	Cat#ab137093; RRID:N/A
Rabbit anti-Phospholipase D1	Cell Signaling technologies	Cat#3832S; RRID:AB_2172256
Rabbit anti-Homer1	Synaptic Systems	Cat#160003; RRID:AB_887730
Guinea pig anti-synapsin 1, 2	Synaptic Systems	Cat#106004; RRID:AB_1106784
Guinea pig anti-synaptophysin 1	Synaptic Systems	Cat#101004; RRID:AB_1210382
Guinea pig anti-Piccolo	Dick et al., 2001	N/A
Alexa Fluor 488 donkey anti-mouse secondary antibody	ThermoFisher Scientific	Cat#A21202; RRID:AB_141607
Alexa Fluor 488 donkey anti-rabbit secondary antibody	ThermoFisher Scientific	Cat#A21206; RRID:AB_141708
Alexa Fluor 488 donkey anti-guinea pig secondary antibody	Dianova/Jackson ImmunoResearch Labs	Cat#706-545-148; RRID:AB_2340472
Cy3 donkey anti-mouse secondary antibody	Dianova/Jackson ImmunoResearch Labs	Cat#715-165-150; RRID:AB_2340813
Cy3 donkey anti-rabbit secondary antibody	Dianova/Jackson ImmunoResearch Labs	Cat#711-165-152; RRID:AB_2307443
Cy3 donkey anti-guinea pig secondary antibody	Dianova/Jackson ImmunoResearch Labs	Cat#706165-148; RRID:AB_2340460
647 donkey anti-mouse secondary antibody	ThermoFisher Scientific	Cat#A31571; RRID:AB_162542
Cy5 donkey anti-rabbit secondary antibody	Dianova/Jackson ImmunoResearch Labs	Cat#711-175-152; RRID:AB_2340607
Cy5 donkey anti-guinea pig secondary antibody	Dianova/Jackson ImmunoResearch Labs	Cat#706-175-148; RRID:AB_2340462
IRDye® 680 Donkey Anti-Mouse secondary antibody	LI-COR	Cat#926-68072; RRID:AB_10953628
IRDye 680RD Goat anti-Rabbit secondary antibody	LI-COR	Cat#926-68071; RRID:AB_10956166
IRDye 800CW Donkey anti-guinea pig secondary antibody	LI-COR	Cat#926-32411; RRID:AB_1850024
Atto 647N- goat anti mouse secondary antibody	Rockland	Cat#610-156-121; RRID:AB_10894200
Atto 647N- goat anti rabbit secondary antibody	Rockland	Cat#611-156-122; RRID:AB_10893043

(*Continued on next page*)

Continued

REAGENT or RESOURCE	SOURCE	IDENTIFIER
Abberior STAR 580- anti mouse secondary antibody	Abberior GmbH	Cat#2-0002-005-1; RRID:AB_2620153
Abberior STAR 580- anti rabbit secondary antibody	Abberior GmbH	Cat#2-0012-005-8; RRID:AB_2810981
Chemicals, Peptides, and Recombinant Proteins		
APV	Tocris	0106 CAS: 79055-68-8
CNQX	Tocris	1045 CAS: 479347-85-8
bafilomycin A1	Merck/Millipore	196000 CAS: 88899-55-2
concanamycin A	Tocris	2656 CAS: 80890-47-7
brefeldin A	Tocris	1231 CAS: 20350-15-6
VU 0155069	Tocris	3575 CAS: 1781834-89-6
Dynole 34-2	Abcam	ab120463 CAS: 1128165-88-7
IPA 3	Tocris	3622 CAS: 42521-82-4
cOmplete ULTRA Tablets	Roche/Merck	05892791001
PhosSTOP	Roche/Merck	PHOSS-RO
mCLING-ATTO647N	Synaptic Systems	710 006AT1
mCLING-DY654	Synaptic Systems	710 006DY1
Critical Commercial Assays		
RT^2 Profiler PCR Array Rat Synaptic Plasticity	QIAGEN	PARN-126Z
RNeasy Plus Mini Kit	QIAGEN	74134
μMACS GFP Isolation Kit	Miltenyi Biotec	130-091-125
μ Columns	Miltenyi Biotec	130-042-701
Deposited Data		
Raw and analyzed data	This paper	N/A
Experimental Models: Cell Lines		
HEK293T (human, embryonic kidney)	ATCC	CRL-3216
Experimental Models: Organisms/Strains		
Rat: Wistar	Charles River	Wistar IGS Rat
Rat: Sprague-Dawley	Charles River	CD® (Sprague Dawley) IGS Rat
Mouse: C57BL/6N	Charles River	C57BL/6NCrl
Mouse: *Ctbp1*^{tm1Sor} (*Ctbp1* KO)	Jackson Lab	(Stock No: 011054)
Oligonucleotides		
CtBP1KD944 shRNA target sequence: GCTTCAACGTCC TCTTCTA	Ivanova et al., 2015	N/A
CtBP1KD467 shRNA target sequence: GCACAGTGGAGA TGCCTAT	Ivanova et al., 2015	N/A
scrambled shRNA sequence: GACTTTACTGCCCCTTACT	Ivanova et al., 2015	N/A
Genotyping primer for CtBP1KO animals ctbp1_common; GAAGTACCAGTACAGGGGACG	Hildebrand and Soriano, 2002	N/A
Genotyping primer for CtBP1KO animals ctbp1_korev; GTTATCGCCGCTCCCGATTCG	Hildebrand and Soriano, 2002	N/A
Genotyping primer for CtBP1KO animals ctbp1_wtrev; CCCCAGCTGACTTGATGTCG	Hildebrand and Soriano, 2002	N/A
Recombinant DNA		
Plasmid: ratio:sypHy	Rose et al., 2013	N/A
Plasmid: syp mOrange2	Egashira et al., 2015	N/A
Lentiviral Plasmid: pCtBP1KD944	Ivanova et al., 2015	N/A
Lentiviral Plasmid: scrambled	Ivanova et al., 2015	
Lentiviral Plasmid: pCtBP1KD467	Ivanova et al., 2015	N/A
Lentiviral Plasmid: pCtBP1KD944 + EGFP-CtBP1	This paper	N/A

(*Continued on next page*)

Continued

REAGENT or RESOURCE	SOURCE	IDENTIFIER
Lentiviral Plasmid: pCtBP1KD944 + YFP-CtBP2(NLS)-CtBP1	This paper	N/A
Lentiviral Plasmid: pCtBP1KD944 + EGFP-CtBP1D355A	This paper	N/A
Lentiviral Plasmid: pCtBP1KD944 + EGFP-CtBP1S147A	This paper	N/A
psPAX2	gift from Didier Trono	Addgene Plasmid #12260
p-CMV-VSV-G	Stewart et al., 2003	Addgene Plasmid #8454
Software and Algorithms		
ImageJ	National Institute of Health	https://imagej.nih.gov/
Openview	Tsuriel et al., 2006	N/A
custom script for STED analysis (MATLAB)	This paper	N/A
custom script for pHluorin analysis (ImageJ)	This paper	N/A
IMOD package	Kremer et al., 1996	https://bio3d.colorado.edu/imod/
Huygens Professional (SVI,15.10.1)	Scientific Volume Imaging	https://svi.nl/Huygens-Professional
Imaris 8.3	Bitplane, Oxford Instruments	https://imaris.oxinst.com/
LightCycler® 480 Software	Roche	https://www.roche.com/
AxoGraph X software	Axograph Scientific	https://axograph.com/
Prism 7 and 8 software	GraphPad Software	https://www.graphpad.com/

LEAD CONTACT AND MATERIALS AVAILABILITY

Further information and requests for resources and reagents can be directed to and will be fulfilled by the Lead Contact, Anna Fejtova (Anna.Fejtova@uk-erlangen.de). Plasmids generated in this study are available from the lead contact upon request and their distribution is regulated by an institutional MTA.

EXPERIMENTAL MODEL AND SUBJECT DETAILS

Animals

Cells and tissues used in this study were obtained from Wistar rats, Sprague-Dawley rats, C57BL/6N mice and *Ctbp1*^{tm1Sor} (*Ctbp1* KO) mouse strain (Hildebrand and Soriano, 2002) backcrossed to C57BL/6N. Animals of both sexes were used. Animal handling was performed according to the regulations of the European Committees Council Directive 86/609/EEC, Landesverwaltungsamt Sachsen-Anhalt, (AZ: T LIN-AF/2009), Berlin state government agency for Health and Social Services and the animal welfare committee of Charité Medical University Berlin, Germany (license no. T 0220/09).

Lentiviral particle production

Lentiviral particles were produced as described previously with slight modifications (Ivanova et al., 2015). HEK293T cells (ATCC CRL-3216) were grown in media containing 10% fetal bovine serum (FBS) to 80% confluence and transfected using the calcium phosphate method (Fejtova et al., 2009) with three vectors: FUGW-based transfer, psPAX2 packaging, and p-CMV-VSV-G pseudotyping vectors (ratio 2:1:1). Cells were incubated for 8 h at 37°C in 5% CO_2 atmosphere, before the FBS medium was replaced by Neurobasal (NB) medium, containing B27, antibiotics, and 0.8 mM glutamine. Virus-containing media was collected at day 3 and 4, passed through 0.45 μm filter and used either directly for transducing primary neurons or stored at −80°C.

Primary cultures and treatments

Primary dissociated hippocampal and cortical cultures from rat embryos and C57BL/6N and *CtBP1* KO neonatal mice were prepared as described in Ivanova et al. (2015) and Lazarevic et al. (2011).

Autaptic cultures from P0-P2 C57BL/6N mice were grown on coverslips with a dotted pattern of astrocytic microislands (Bekkers and Stevens, 1991). To grow neurons individually, 0.15% agarose solution was spread on 30 mm coverslips. Coating solution containing collagen and poly-D-lysine in acetic acid was stamped onto the agarose, thus creating small islands of substrate with a diameter of about 100 μm. Hippocampi were dissected out and digested with 25 U/ml of papain for 60 min at 37°C. After papain inactivation, hippocampi were mechanically dissociated in Neurobasal-A medium containing B-27, Glutamax and penicillin/streptomycin. To obtain a desirable distribution of neurons, astrocytes and neurons were plated onto the coverslips with a density of 50000 and 3000 cells/coverslip, respectively. To knock down CtBP1, neurons were infected 24 hours later with lentiviruses expressing

scrambled, shRNA against CtBP1 or the rescue constructs EGFP-CtBP1 and YFP-CtBP2(NLS)-CtBP1. Experiments were performed on DIV14 (electrophysiological recordings) or DIV16-21 (fixed and live-cell imaging).

Hippocampal neurons were co-transfected with syp mOrange2 and a plasmid expressing CtBP1 scr, CtBP1KD944 or CtBP1KD944 along with shRNA-resistant EGFP-CtBP1, EGFP-CtBP1D355A or YFP-CtBP2(NLS)-CtBP1 at DIV6 using Lipofectamine 2000 (Thermo Fisher Scientific) as recommended by the manufacturer. The neurons were used for live imaging 8 to 10 days after the transfection.

For the treatments, the following drugs were used: d-(-)-2-amino-5-phosphonopentanoic acid (APV, 50 μM; Tocris), 6-cyano-7-nitroquinoxaline-2,3-dione disodium (CNQX, 10 μM; Tocris), bafilomycin A1 (1 μM, Merck/Millipore), folimycin/concanamycin A (80nM, Tocris), brefeldin A (2.5 μM, Tocris), VU 0155069 (PLD1 inhibitor, 1 μM, Tocris). Neurons were pre-treated with these inhibitors for 5 minutes before imaging and the inhibitors were kept in the imaging buffer during the whole imaging assay. IPA 3 (50 μM, Tocris) was applied for 1h before the cells were collected or lysed for western blotting. The inhibitors of dynamin, Dynole 34-2 (30 μM, Abcam) was applied for 1h during Syt1 Ab-CypHer uptake. The fixable endocytosis marker mCLING (ATTO647N-labeled in Figures 6G and 6H and DY654-labeled in Figures 6E and 6F; 1:100, Synaptic Systems) was applied to neurons in extracellular solution containing 50 μM APV and 10 μM CNQX, for 2 min before cells were stimulated with 200 AP at 40 Hz. To eliminate unspecific labeling neurons were washed three times with extracellular solution and fixed within 30 s after stimulation with a mixture of 4% paraformaldehyde (PFA) and 0.2% glutaraldehyde, as recommended by the manufacturer.

METHOD DETAILS

Antibodies

The following primary antibodies were used in this study: **Mouse antibodies against**: CtBP1 (immunocytochemistry (ICC) 1:1,000, western blotting (WB) 1:5,000, BD Biosciences, 612042), CtBP2 (WB 1:2000 BD Biosciences, 612044) synaptotagmin1 lumenal domain Oyster 550 or CypHer5E-labeled (ICC 1:200, Synaptic Systems, 105311 and 105311CpH), rab5 (ICC 1:500, Synaptic Systems, cells stained with this antibody were fixed with ice-cold methanol for 10 min, followed by rehydration in PBS for 20 min, 108011), rab7 (ICC 1:1,000, Abcam, ab50533), phosphoserine/threonine (WB 1:1000, BD Biosciences, 612548), GluA Oyster 550-labeled (ICC 1:200, Synaptic Systems, 182411 C3), α-tubulin (WB 1:1000, Sigma Aldrich); **Rabbit antibodies against**: CtBP1 (ICC 1:1,000, WB 1:1,000, Synaptic Systems, 222002), GFP (ICC 1:1,000, WB 1:5,000, Abcam, ab 6556), SV2B (ICC 1:200, Synaptic Systems, 119103), GAPDH (WB 1:3000, Abcam, ab37168), synaptotagmin1 lumenal domain Oyster 550-labeled (ICC 1:200, Synaptic Systems, 105103C3), synaptotagmin 1 lumenal domain (WB 1:1000, Synaptic Systems, 105102), dynamin1 (ICC 1:1000, Abcam, ab3456), rab22a (ICC 1:1000, Abcam, ab137093), Phospholipase D (WB 1:1000, Cell Signaling technologies, 3832S), Homer1 (ICC 1:500, Synaptic Systems, 160003); **Guinea pig antibodies against**: synapsin 1, 2 (ICC 1:1,000, Synaptic Systems, 106004), synaptophysin 1 (ICC 1:1,000, Synaptic Systems, 101004), Piccolo (WB 1:2000, Dick et al., 2001).

The following secondary cross-adsorbed antibodies were used in this study: Alexa 488- (ICC: 1:1,000), Cy3-(ICC: 1:1,000), Cy5-(ICC: 1:2,000), Alexa 680- (WB 1:20,000) conjugated whole IgGs against mouse, rabbit and guinea pig were obtained from Invitrogen/Mol. Probes, IRDye 800CW (WB 1:20,000) and Atto 647N (1:500, 610-156-121 and 611-156-122) from Rockland and Abberior STAR 580 (1:100, 2-0002-005-1 and 2-0012-005-8) from Abberior GmbH.

DNA constructs

EGFP-tagged CtBP1 was generated by cloning the sequence for CtBP1-S into pEGFPC vector. Subsequently, the DNA cassette containing EGFP-CtBP1 was shuttled into FUGW H1 lentiviral vector (Leal-Ortiz et al., 2008), replacing EGFP coding sequence. The shRNAs against CtBP1 and YFP-CtBP2(NLS)-CtBP1 constructs were reported previously (Ivanova et al., 2015; Verger et al., 2006). All point mutations, including the silent point mutations for the rescue experiments, were introduced by inverse PCR using primers containing the mutations and CtBP1-S coding sequence cloned in pBluescriptII SK-(AgilentTechnologies). The ratio:sypHy construct and syp mOrange2 used in this study were reported in Lazarevic et al. (2017); Rose et al. (2013), and Egashira et al. (2015), respectively. All constructs were verified by sequencing.

Ultrastructural analysis

Organotypic hippocampal slice cultures from *Ctbp1* KO and WT littermates were prepared at postnatal day 0 and were cryo-fixed after 4-5 weeks *in vitro* under cryo-protectant conditions (20% bovine serum albumin in culture medium) using the High Pressure Freezing device HPM100 (Leica), and cryo-substituted in Freeze Substitution Processor EM AFS2 (Leica) according to previously published protocols (Imig and Cooper, 2017; Imig et al., 2014). For 2D analyses of synaptic morphology, electron micrographs were acquired from 60 nm-thick plastic sections with a transmission electron microscope (Zeiss LEO 912-Omega) operating at 80 kV. For 3D electron tomographic analysis of docked SV, 200 nm-thick plastic sections were imaged in a JEM-2100 transmission electron microscope (JEOL) operating at 200 kV. SerialEM (Mastronarde, 2005) was used to acquire single-axis tilt series (−60°/-55° to ± 55°/ ± 60°; 1° increments) at 25,000 fold magnification with an Orius SC1000 camera (Gatan, Inc.). Tomograms reconstructed from tilt series using the IMOD package (Kremer et al., 1996) had a voxel size of x,y,z = 1.82 nm. Tomogram acquisition and analyses were performed blindly. Quantifications were done manually using ImageJ (National Institutes of Health). The smallest SV distances from the outer leaflet of the SV membrane to the inner leaflet of the AZ plasma membrane were measured using the straight line tool of

the ImageJ software. Only SVs observed to be in physical contact at their midline with the presynaptic membrane were considered docked (0-2 nm distance). The mean SV diameter was calculated from the area of the SV measured at its midline to the outer leaflet of the SV membrane using the elliptical selection tool of ImageJ.

For illustrative purposes, images depicting tomographic sub-volumes represent an overlay of seven consecutive tomographic slices produced using the slicer tool of the 3dmod software of the IMOD software package to generate an approximately 13 nm thick sub-volume.

Quantitative real-time PCR

Quantitative real-time PCR was performed as described in Ivanova et al. (2015). Total RNA was extracted from primary cortical cultures (DIV16) superinfected on the day of plating with lentiviral particles driving the expression of scrambled, shRNA944 and YFP-CtBP2(NLS)-CtBP1, using RNeasy Plus Mini Kit (QIAGEN) and following the instructions of the manufacturer. The transcript levels of BDNF and Arc were analyzed by a customized version of Rat Synaptic Plasticity RT^2 Profiler PCR Array (QIAGEN). To calculate the expression of BDNF and Arc in relation to a reference gene we used ΔΔCP method. We used the 'second derivative maximum analysis' method, available in the software of Roche LightCycler480, to determine the crossing point (CP) of the PCR. The expression of lactate dehydrogenase A was used as a reference to calculate the relative mRNA levels of BDNF and Arc.

Biochemical experimental work

Cortical neurons with cell density 10 million per 75-cm2 flask were superinfected with lentiviral particles, driving the expression of EGFP-CtBP1. Cells (DIV16) were lysed in 10mM Tris–HCl, 150mM NaCl, 2% SDS, 1% deoxycholate and 1% Triton X-100 containing complete protease inhibitors (Roche), and PhosStop (Roche) and co-immunoprecipitations were performed using MicroMACS anti-GFP MicroBeads and MicroColumns (Miltenyi Biotec) according to the instructions from the manufacturer.

Crude synaptosomal fraction (P2) was prepared as follows: First, cell or mouse brain homogenates were prepared in HEPES-buffered sucrose (4 mM HEPES pH 7.4, 0.32 M sucrose) and centrifuged at 1000 x g for 10 min to pellet the nuclear fraction (P1). The supernatant was then centrifuged at 12000 g for 20 min to give the crude synaptosomal pellet (P2). The crude synaptosomal fraction (P2) was lysed in 10 mM Tris–HCl, 150mM NaCl, 2% SDS, 1% deoxycholate and 1% Triton X-100 containing complete protease inhibitors (Roche), and PhosStop (Roche) and further subjected to IP or western blotting.

Protein samples were separated on 5%–20% Tris-glycine gels, or 3.5%–8% Tris-acetate gels as described previously (Ivanova et al., 2015) or on 10% (Bio-Rad TGX-Stain free gels) and blotted onto Millipore Immobilon FL PVDF membranes by tank or semidry blotting. Immunodetection was performed on Odyssey Infrared Scanner (LI-COR). For the quantification of the immunoblots the integrated density (ID) of signals was measured using ImageJ by setting rectangular ROIs with identical size around or using Image Studio Software (LI-COR). Samples of each experimental group were always loaded and quantified on the same membrane. TCE total protein stain was used for normalization in Figure 1B. In Figure S2A, GAPDH or α-tubulin were used for normalization in homogenates and P2 fraction, respectively. The values for ID of CtBP1 (Figures 7A–7D) were normalized to the corresponding expression levels of the two proteins in each experimental group. The antibodies used for immunodetection and the molecular weight of the markers are indicated in the figures.

Microscopy and image analysis

Immunostaining of neurons was performed as described in Lazarevic et al. (2011). For quantifications, identical antibodies solutions were used for all coverslips from the same experiment. For the co-localization analysis, neurons were silenced with APV and CNQX for 10 minutes, in order to minimize the effect of the ongoing activity on the variance between synapses and then stimulated with 200 AP at 40 Hz. Cells were fixed within 30 s after the end of stimulation.

Staining with synaptotagmin 1 antibody (Syt1 Ab uptake) was performed by incubating the cells with fluorescently-labeled primary antibody dissolved in extracellular solution, containing 119 mM NaCl, 2.5 mM KCl, 2 mM CaCl2, 2 mM MgCl2, 30 mM glucose, and 25 mM HEPES, pH 7.4 for 30 min at 37°C (Lazarevic et al., 2011) before fixation. For the imaging with CypHer5E-labeled anti-synaptotagmin1 antibody, cells were incubated with the antibody diluted in a buffer containing 120 mM NaCl, 5 mM KCl, 2 mM MgCl2, 2 mM CaCl2, 10 mM glucose, and 18 mM NaHCO3, pH 7.4 for 2-3 hours at 37°C prior imaging.

Epifluorescence images were acquired on a Zeiss Axio Imager A2 microscope with Cool Snap EZ camera (Visitron Systems) controlled by VisiView (Visitron Systems GmbH) software.

Confocal images in Figure S3A were acquired on a Leica SP5 confocal microscope. The format of the images was 2048x2048 pixels display resolution, 8 bit dynamic range, for acquisition 63x objective, NA 1.40 and 2x optical zoom were used, which results in a voxel size of approximately 50 nm.

Dual-color STED images (1024x1024 pixels display resolution, 8 bit dynamic range) were acquired on a Leica TCS SP8-3X gated STED microscope using a HC APO CS2 100x objective, NA 1.40, and 5x optical zoom, corresponding to a voxel size of approximately 23 nm. 16 times line averaging was applied on frames acquired at a scan speed 600 Hz. The built-in pulsed white light laser of the setup was used to excite Abberior STAR 580 and Atto 647N at 561 nm and 650 nm, respectively. The detection was done at 580-620 nm for Abberior STAR 580 and 660-730 nm for Atto647N. Both dyes were depleted using a pulsed 775 nm depletion laser. Time-gated detection of 0.5-1 ns to 6 ns was set for both STED channels. All raw data were subsequently deconvolved using the calculated point spread function (PSF) of the system and the Classic Maximum Likelihood Estimation (CMLE) algorithm with Huygens

Professional (SVI,15.10.1). In brief, after an automatic background correction, the signal to noise ratio was set to 15 and the optimized iteration mode of the CMLE was run until a quality threshold of 0.05 was reached. The deconvolved datasets were corrected for a chromatic aberration in z, using the Chromatic Aberration Corrector (CAC) in Huygens.

The co-localization analysis was performed on the deconvolved STED stacks using Imaris 8.3 (Bitplane, Oxford Instruments). To detect punctate staining as spots Imaris spot detection algorithm was applied as follows: the sensitivity for the detection of the spots in each channel was determined by an automatically generated threshold and the spots diameter was set to 0.06 μm. The distances between the spots in the two channels were measured using a customized version of the Imaris XTension Spots Colocalize, which determines the co-localization between the spots within a user-defined distance (1 μm) and bins the data into several bins with equal width (100 nm).

For quantifications, the same detector settings were used for all coverslips quantified in one experiment. From each culture, images from at least two different coverslips were acquired and quantified to minimize experimental variability. The nuclear fluorescence was assessed as established before (Ivanova et al., 2015). ImageJ (NIH) and OpenView software (Tsuriel et al., 2006) were used for quantitative immunofluorescence analysis. After removing the background by threshold subtraction in ImageJ, synaptic puncta were defined with OpenView software by setting rectangular regions of interest (ROI) with identical dimensions around local intensity maxima in the channel with staining for synapsin or any of the other synaptic markers that were used (GluA, homer1, synaptophysin, SV2B). Mean immunofluorescence (IF) intensities were measured in the synaptic ROIs in all corresponding channels using the same software and normalized to the mean IF intensities of the control group for each of the experiments. The number of synapses per unit of dendrite length was determined as follows: First synapsin puncta along 30 μm of proximal dendrite, was detected using Find Maxima function in ImageJ, by setting the same noise tolerance to all images quantified in one experiment; Mean IF intensities of GluA were measured in circular ROIs set around the local intensity maxima in the image with synapsin staining; The number of GluA puncta co-localizing with synapsin was calculated by applying an identical intensity threshold for GluA detection between the different conditions within an experiment.

pHluorin imaging and analysis

The pHluorin imaging was performed with hippocampal cultures DIV16 to 20, transduced with lentiviral particles on the day of plating.

The coverslips were removed from the cell culture plates and mounted in an imaging chamber (Warner instruments), supplied with a pair of platinum wire electrodes, 1 cm apart, for electrical stimulation. The imaging was performed at 26°C in extracellular solution, containing 119 mM NaCl, 2.5 mM KCl, 25 mM HEPES pH7.4, 30 mM glucose, 2 mM MgCl2 and 2 mM CaCl2, 10 μM 6-cyano-7-nitroquinoxaline-2,3-dione disodium (CNQX, Tocris) and 50 μM d-(-)-2-amino-5-phosphonopentanoic acid (APV, Tocris), on inverted microscope (Observer. D1; Zeiss-as described above) equipped with an EMCCD camera (Evolve 512; Photometrics) controlled by MetaMorph Imaging (MDS Analytical Technologies) and VisiView (Visitron Systems GmbH) software, using 63x objective. EGFP ET filter set (exciter 470/40, emitter 525/50, dichroic 495 LP, Chroma Technology Corp.) and Cy5 ET filter set (exciter 620/60, emitter 700/75, dichroic 660 LP, Chroma Technology Corp.) were used for imaging of the pHluorin and CypHer5E, respectively. Cultures were stimulated with a train of 40 or 200 action potentials (1 ms, constant voltage pulses) at 5, 20 or 40 Hz using S48 stimulator (GRASS Technologies). The alkaline trapping method was used for quantification of the recycling vesicle pools. In brief, the stimulation of sypHy expressing neurons was done in presence of bafilomycin A1 (1 μM, Merck/Millipore), a specific inhibitor of the vesicular V-type ATPase. Exocytosis of RRP was triggered by delivering of 40 AP at 20 Hz. Following a 2 min break after the end of the first train of stimuli TRP was released by stimulation with 200 AP at 20 Hz. The relative sizes of RRP and TRP were determined as fractions of the total sypHy-expressing pool measured after addition of alkaline imaging buffer (60 mM NaCl in the extracellular solution was replaced with 60 mM NH_4Cl). Fluorescent images were acquired at 1 Hz (Figure 1I) and 10 Hz (Figures 1F, 1J, 1K, 4E, 6A–6D, S2C, S2G, and S4). Imaging of hippocampal neurons transfected with syp mOrange2 (Figure 4C) was performed in a modified extracellular solution (136-mM NaCl, 2.5 mM KCl, 2 mM $CaCl_2$, 1.3 mM $MgCl_2$, 10 mM glucose, and 10 mM HEPES, 10 μM CNQX, 50 μM APV, pH 7.4) on inverted Zeiss Axio Observer.Z1 epifluorescence microscope, equipped with Zeiss AxioCam 506 camera controlled by ZEISS ZEN 2 software, using EC Plan-Neofluar 40x oil immersion objective (NA 1.3) and a DsRED filter set (exciter 538-562, beam splitter 570, emitter 570-640). Cultures were stimulated with a train of 200 AP delivered at 20 Hz (100 mA, 1 ms pulse width) and fluorescent images were acquired at 0.5 Hz. Synaptic puncta responding to stimulation were identified by subtracting an average of the first several frames of the baseline from an average of several frames at the end of stimulation. The mean IF intensities were measured in ROIs with an identical size, placed automatically over each responding synapse using a self-written macro in ImageJ. The data traces were determined after removing the background by threshold subtraction and correction for bleaching, calculated from the bleaching of unresponsive boutons from the same coverslip. The half times for endocytosis (t1/2) were determined by applying a single exponential fit to the decay phases of the data traces using GraphPad Prism5 and the following equation: Ft = Fstim*exp(-t/tau), t1/2 = ln(2)*tau, where Fstim is the fluorescence intensity at the end of stimulation and tau is the time constant for endocytosis.

Electrophysiology

Whole-cell voltage clamp recordings were performed between 14 and 18 days *in vitro* (DIV) in autaptic neurons at room temperature. Ionic currents were acquired using a Digidata 1440A digitizer and a Multiclamp 700B amplifier under the control of Clampex X software (Axon instrument). Series resistance was set at 70% and only neurons with series resistances below 10 MΩ were selected. Data

were recorded at 10 kHz and low-pass filtered at 3 kHz. Borosilicate glass pipettes with a resistance around 3 MΩ were used and filled with an intracellular solution containing (in mM): 136 KCl, 17.8 HEPES, 1 EGTA, 4.6 $MgCl_2$, 4 Na_2ATP, 0.3 Na_2GTP, 12 phosphocreatine, and 50 U/ml phosphocreatine kinase; 300 mOsm; pH 7.4. Autaptic neurons were continuously perfused with standard extracellular solution composed of (in mM): 140 NaCl, 2.4 KCl, 10 HEPES, 10 glucose, 2 $CaCl_2$, 4 $MgCl_2$; 300 mOsm; pH 7.4. Spontaneous release was measured by recording mEPSC for 30 s at a holding potential of −70 mV in the presence of 3 mM kynurenic acid to detect false positive events and for the equal amount of time in extracellular solution. Data were filtered at 1 kHz and analyzed using template-based miniature event detection algorithms implemented in the AxoGraph X software. Action potential-evoked release EPSCs were elicited by 2 ms somatic depolarization from −70 to 0 mV. To estimate the readily-releasable pool (RRP) size, 500 mM hypertonic sucrose added to standard extracellular solution, was applied for 5 s using a fast-flow system (Pyott and Rosenmund, 2002). For vesicular release probability (Pvr) calculations, the ratio of EPSC charge to RRP charge was determined. Short-term plasticity was examined either by evoking 2 unclamped AP with 25 ms interval (40 Hz) or a train of 50 AP at an interval of 100 ms (10 Hz). All electrophysiological data were analyzed offline using Axograph X (Axograph Scientific).

QUANTIFICATION AND STATISTICAL ANALYSIS

All quantitative results are given as means ± standard errors of the mean (SEM) and normalized to the values of control. Statistical analyses were performed with Prism 7 and 8 (GraphPad Software, Inc.). The sample sizes (n numbers) were adjusted based on published studies using similar methodology. n numbers correspond to the number of cells (fixed cell imaging and electrophysiology experiments), individual coverslips (live cell imaging experiments), synaptic profiles (EM data), number of independent immunoprecipitations (IP) or samples from independent animals (WB) and are indicated for each group in the graphs. In the graphs comparisons with the control are indicated above each box and, comparisons between the conditions are given as horizontal bars. The statistical tests were chosen after the distribution of the datasets was explored. The scoring and the statistical tests used to compute the P values and the numeric values of all results are specified in the Table S3. Significance is indicated using asterisks: nsP > 0.05, *p < 0.05, **p < 0.01, ***p < 0.001, **** p < 0.0001.

DATA AND CODE AVAILABILITY

Requests for data and the scripts used for the main steps of the analysis of the pHluorin and STED data should be directed to the Lead Contact Anna Fejtova and will be made available upon reasonable request.

Cell Reports, Volume *30*

Supplemental Information

CtBP1-Mediated Membrane Fission Contributes to Effective Recycling of Synaptic Vesicles

Daniela Ivanova, Cordelia Imig, Marcial Camacho, Annika Reinhold, Debarpan Guhathakurta, Carolina Montenegro-Venegas, Michael A. Cousin, Eckart D. Gundelfinger, Christian Rosenmund, Benjamin Cooper, and Anna Fejtova

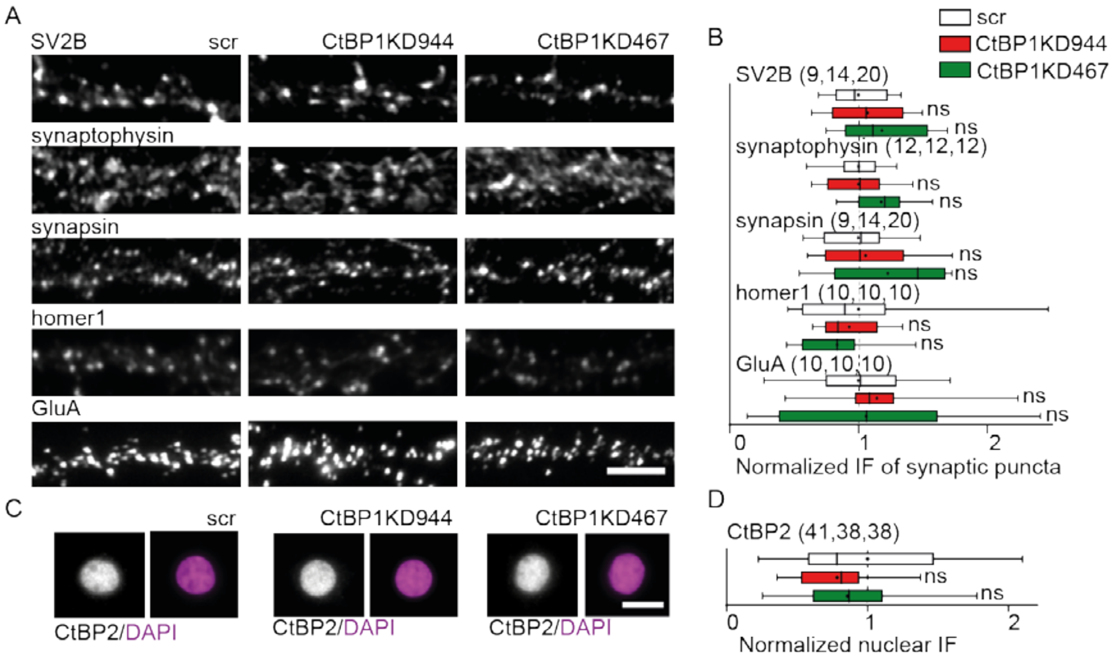

Figure S1 (Related to Figure 1)

Knock down of CtBP1 does not affect the overall expression of synaptic proteins and CtBP2

A) Synaptic abundance of pre- (SV2B, synapsin, synaptophysin) and post-synaptic markers (homer1, GluA) does not change in CtBP1KD neurons.
B) Quantification of the effects shown in A)
C) Nuclear CtBP2 does not change in CtBP1KD neurons.
D) Quantification of the effects shown in C)

Scale bar is 5 µm in A, and 10 µm in C. In the plots the interquartile range and median are depicted as boxes, minimal and maximal values as whiskers and + indicates mean.

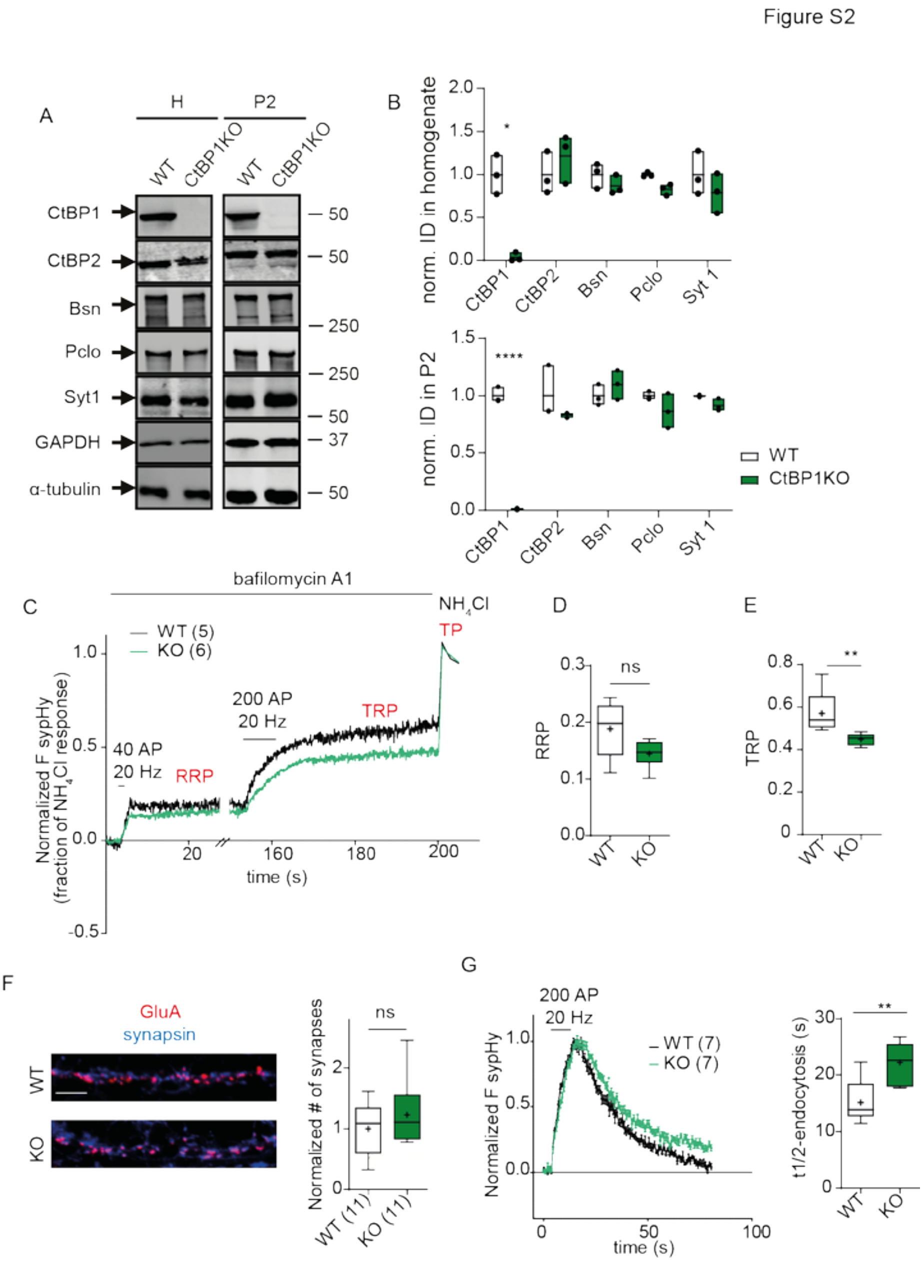

Figure S2 (Related to Figure 1)

***Ctbp1* KO synapses have a reduced rate of SV endocytosis and a lower number of release-competent vesicles.**

A) Immunoblot detection of synaptic proteins in brain homogenates (H) and crude synaptosomes (P2) from WT and *CtBP1*-/- mice. GAPDH and α-tubulin are loading controls.

B) Quantification of the effects shown in A)

C) Average sypHy-fluorescence traces reporting SV pool sizes from neurons derived from WT and *Ctbp1*-/- mice.

D) The mean values of RRP in WT and *Ctbp1*-/- did not differ significantly.

E) Quantification of TRP size in WT and *Ctbp1*-/- .

F) Neurons prepared from *Ctbp1*-/- animals and their WT siblings stained with an anti synapsin Ab, to label presynaptic terminals and pan anti GluA Ab to label postsynapses. Number of co-localizing synapsin and GluA puncta was slightly but not significantly increased in KO compared to control. The overlays are shown in the indicated colors. Scale bar: 5µm.

G) Peak-normalized sypHy responses to 200 AP at 20Hz. The half times: t1/2 of endocytosis (bar graph) were smaller in WT neurons compared to *Ctbp1*-/- .

In the plots the interquartile range and median are depicted as boxes, minimal and maximal values as whiskers and + indicates mean. Significance is indicated using asterisks: nsP > 0.05, ∗p < 0.05, ∗∗p < 0.01, ∗∗∗∗ p < 0.0001.

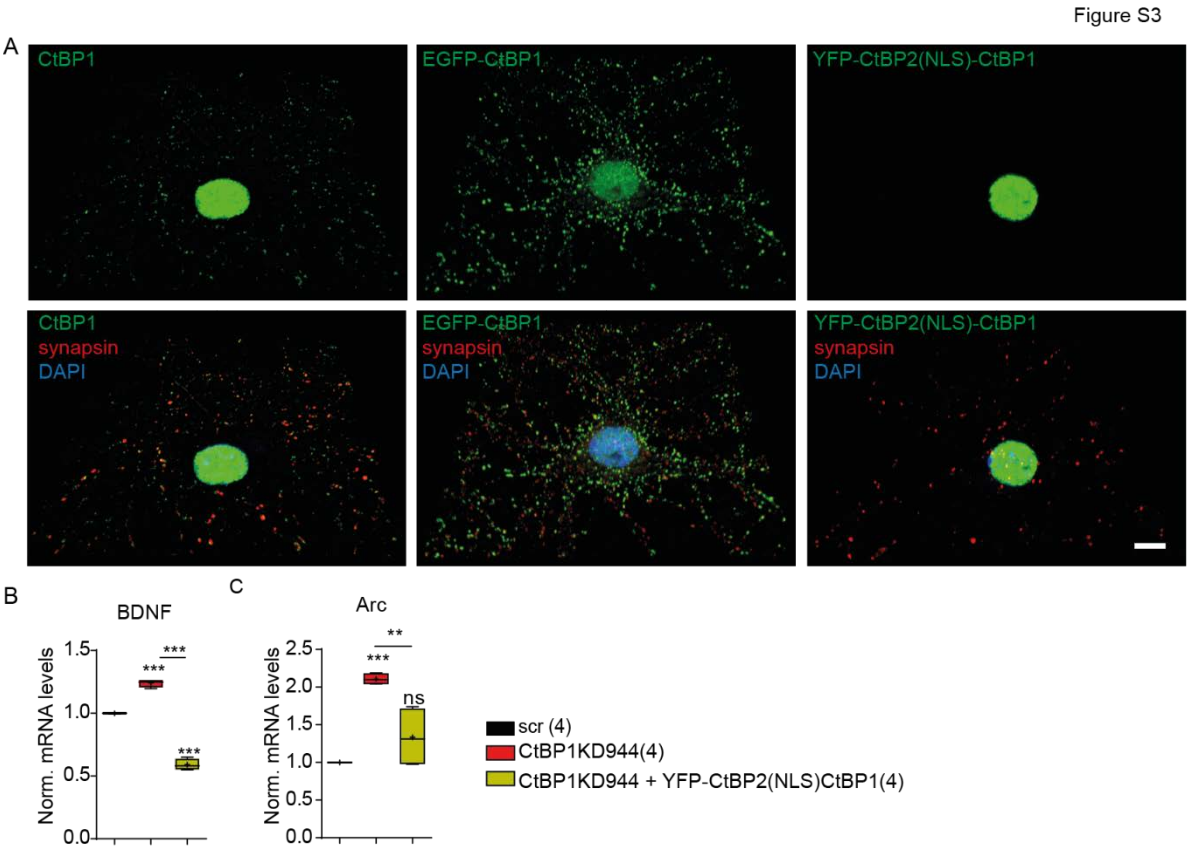

Figure S3 (Related to Figure 3 and 4)

Expression of YFP-CtBP2(NLS)-CtBP1 reverts the effect of CtBP1KD944 on gene expression.

A) Perspective views of 3D reconstructions of hippocampal neurons showing the synapto-nuclear distribution of the endogenous CtBP1 and the expressed rescue variants. Synapsin staining labels presynaptic terminals; DAPI labels nuclei. Note that EGFP-CtBP1 shows a decreased nuclear and an increased synaptic localization, whereas YFP-CtBP2(NLS)-CtBP1 is expressed only in the nucleus. For better visualization several EGFP-CtBP1-positive spots were removed from the planes above the nucleus. Overlays are shown in the indicated colors. Scale bar: 7µm.

B and C) YFP-CtBP2(NLS)-CtBP1 counteracts the increased expression of BDNF and Arc in CtBP1KD944 neuronal cultures.

In the plots the interquartile range and median are depicted as boxes, minimal and maximal values as whiskers and + indicates mean. In the graphs comparisons with the control are indicated above each box and, comparisons between the conditions are given as horizontal bars. Significance is indicated using asterisks: $**p < 0.01$, $***p < 0.001$.

Figure S4

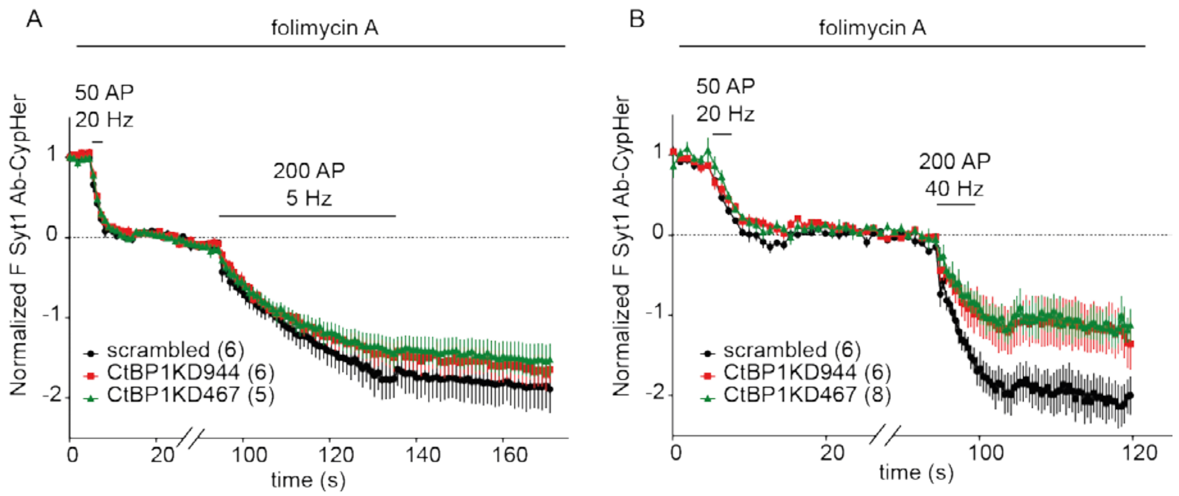

Figure S4 (Related to Figure 4)

Frequency-dependent short-term synaptic depression at CtBP1-deficient synapses

A) and B) Average Syt1 Ab-CypHer responses to 50 AP at 20 Hz (a reference response), followed by a 60s rest period and 200 AP at 5 Hz (A) or 40 Hz (B) in the presence of 80 nM folimycin. The traces were normalized to the amplitudes of the reference response. KD of CtBP1 reduces the fluorescence responses to 200 AP at 5 Hz and even more pronouncedly at 40 Hz.

Data points in curves are depicted as means and SEM.

Figure S5

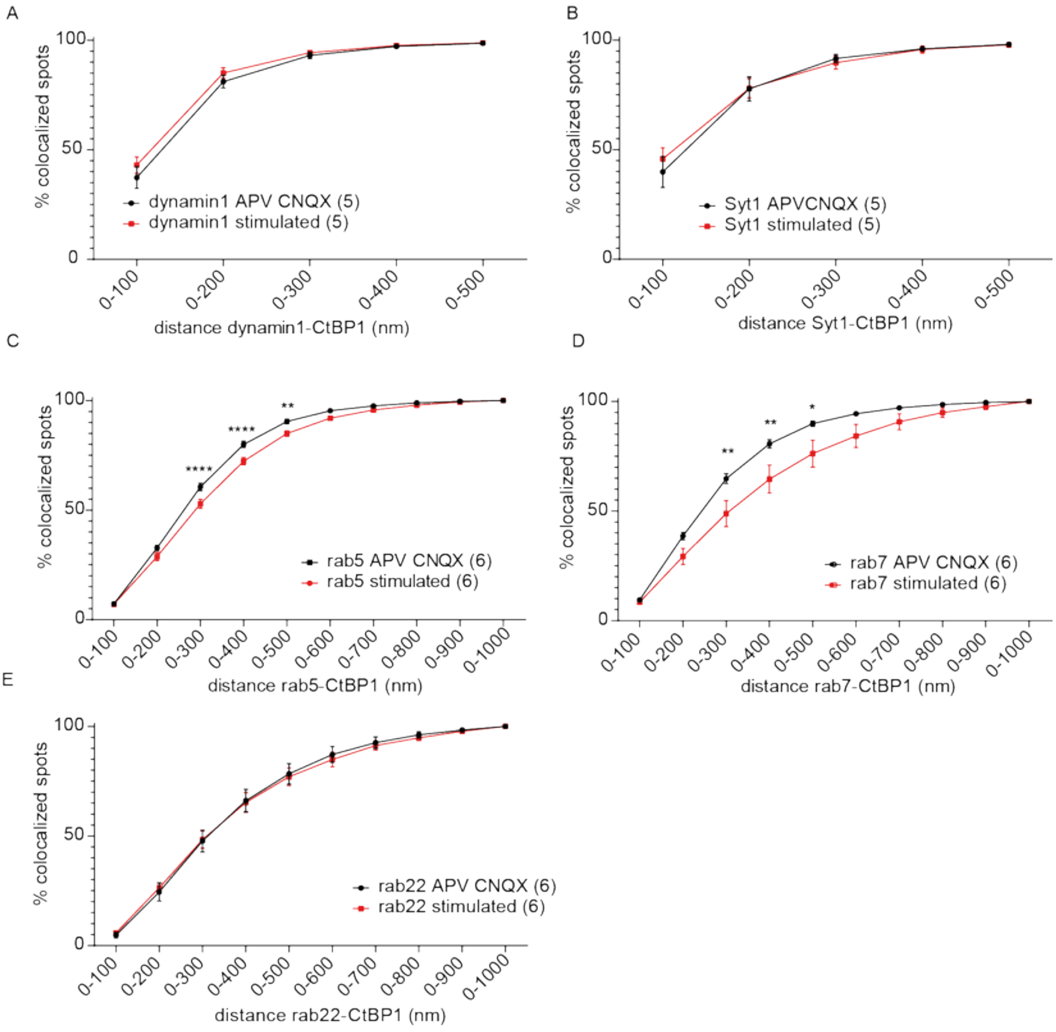

Figure S5 (Related to Figure 5)

Effect of synaptic stimulation on the co-localization of CtBP1 with the endocytic markers dynamin1, rab5, rab7, rab22 and the SV protein Syt1.

A - E) Cumulative plots showing the % of dynamin1, rab5, rab7, rab22 and Syt1 puncta co-localizing with CtBP1 in control (treated with 50µM APV and 10µM CNQX for 10 min) and stimulated (200AP at 40Hz) neurons, binned according to the distance to CtBP1 labelled spots.

Data points are depicted as means and SEM. Significance is indicated using asterisks: ∗p < 0.05, ∗∗p < 0.01, ∗∗∗∗ p < 0.0001.

Table S1: Quantitative analysis of synaptic ultrastructure (Related to Figure 2)

2D EM Analysis of Synaptic Morphology

	**WT (N=3, n=159)**	**KO (N=4, n=146)**	
# of SVs per profile	80.72 ± 3.244	89.21 ± 3.721	P = 0.098
terminal area (x 0.01 µm^2)	40.38 ± 1.182	41.19 ± 1.303	P = 0.845
# SVs / 0.01 µm^2 terminal area	1.993 ± 0.054	2.159 ± 0.064	P = 0.065
PSD length (nm)	373.7 ± 9.261	379.4 ± 9.421	P = 0.627
# of endosomes / terminal	0.843 ± 0.077	0.726 ± 0.082	P = 0.140
# of LDCVs / terminal	0.151 ± 0.034	0.24 ± 0.043	P = 0.083

N, number of animals; n, number of synaptic profiles; SV, synaptic vesicle; PSD, postsynaptic density; LDCV, large dense-core vesicle. (red P-values = Mann-Whitney test, black P-values = unpaired t-test)

3D Electron Tomographic Analysis of Synaptic Vesicle Pools

	**WT (N=3, n=26)**	**KO (N=4, n=25)**	
# SVs within 0-2 nm of AZ	0.605 ± 0.092	0.876 ± 0.117	P = 0.075
# SVs within 0-5 nm of AZ	0.797 ± 0.109	1.213 ± 0.142	*P = 0.043
# SVs within 0-40 nm of AZ	1.821 ± 0.12	2.496 ± 0.168	**P = 0.002
# SVs within 0-100 nm of AZ	5.876 ± 0.267	7.307 ± 0.382	**P = 0.003
# SVs within 0-200 nm of AZ	14.65 ± 0.817	15.31 ± 0.811	P = 0.572
# SVs within 5-10 nm of AZ	0.214 ± 0.041	0.292 ± 0.07	P = 0.621
# SVs within 10-20 nm of AZ	0.264 ± 0.058	0.162 ± 0.037	P = 0.354
# SVs within 20-30 nm of AZ	0.213 ± 0.051	0.363 ± 0.069	P = 0.072
# SVs within 30-40 nm of AZ	0.345 ± 0.052	0.465 ± 0.07	P = 0.170
# SVs within 40-50 nm of AZ	0.531 ± 0.053	0.596 ± 0.081	P = 0.503
# SVs within 50-100 nm of AZ	3.54 ± 0.196	4.215 ± 0.245	*P = 0.036
# SVs within 100-150 nm of AZ	4.408 ± 0.331	4.175 ± 0.251	P = 0.579
# SVs within 150-200 nm of AZ	4.34 ± 0.328	3.827 ± 0.291	P = 0.249
AZ area (nm^2)	40.900 ± 1.775	44.240 ± 2.276	P = 0.569
SV diameter (SVs within 0-200 nm of AZ)	44.95 ± 0.347	45.77 ± 0.38	P = 0.114
SV diameter (SVs within 0-100 nm of AZ)	44.98 ± 0.381	45.82 ± 0.426	P = 0.15

N, number of animals; n, number of tomograms; SV, synaptic vesicle; AZ, active zone. SV numbers within a certain distance of the AZ are normalized to 0.01 µm^2 of AZ area. Values indicate mean ± SEM. (red P-values = Mann-Whitney test, black P-values = unpaired t-test)

	**WT (n=63)**	**KO (n=100)**	
SV diameter (docked SVs, 0-2 nm of AZ)	44.17 ± 0.64	46.08 ± 0.485	*P = 0.012

n, number of docked SVs averaged over all tomograms of a given genotype

Table S2: Electrophysiological analysis of autaptic cultures from CtBP1944KD and scr and upon expression of selective synaptic or nuclear rescue constructs (Related to Figure 3)

<table>
<tr><th></th><th>SC</th><th>Kruskal-Wallis test</th><th>CtBP1KD944</th><th>Kruskal-Wallis test</th><th>EGFP-CtBP1</th><th>Kruskal-Wallis test</th><th>YFP-CtBP2(NLS)-CtBP1</th><th>Kruskal-Wallis test</th></tr>
<tr><td rowspan="3">mEPSC charge (fC)</td><td rowspan="3">110.5 ± 4.2 (n=69/5)</td><td>CtBP1KD944 P>0.99</td><td rowspan="3">104.4 ± 4.1 (n=70/5)</td><td>SC P>0.99</td><td rowspan="3">119.4 ± 9.8 (n=64/5)</td><td>SC P>0.99</td><td rowspan="3">110.3 ± 4.1 (n=62/5)</td><td>SC P>0.99</td></tr>
<tr><td>EGFP-CtBP1 P>0.99</td><td>EGFP-CtBP1 P>0.99</td><td>CtBP1KD944 P>0.99</td><td>CtBP1KD944 P>0.99</td></tr>
<tr><td>YFP-CtBP2(NLS)-CtBP1 P>0.99</td><td>YFP-CtBP2(NLS)-CtBP1 P>0.99</td><td>YFP-CtBP2(NLS)-CtBP1 P>0.99</td><td>EGFP-CtBP1 P>0.99</td></tr>
</table>

EPSC Charge (pC)	35.4 ± 4.5 (n=77/5)	CtBP1KD944 P=0.0018	55.2 ± 5.9 (n=72/5)	SC P=0.0018	78.1 ± 8.5 (n=62/5)	SC <0.0001	51.3 ± 6.2 (n=63/5)	SC P=0.072
		EGFP-CtBP1 P<0.0001		EGFP-CtBP1 P=0.4137		CtBP1KD944 P=0.4137		CtBP1KD944 P>0.99
		YFP-CtBP2(NLS)-CtBP1 P=0.072		YFP-CtBP2(NLS)-CtBP1 P>0.99		YFP-CtBP2(NLS)-CtBP1 P=0.0436		EGFP-CtBP1 P=0.0436
Pvr (%)	7.0 ± 0.5 (n=73/5)	CtBP1KD944 P<0.0001	15.8 ± 0.9 (n=64/5)	SC P<0.0001	14.2 ± 1.1 (n=52/5)	SC P<0.0001	11.6 ± 1.0 (n=62/5)	SC P>0.006
		EGFP-CtBP1 P<0.0001		EGFP-CtBP1 P>0.999		CtBP1KD944 P>0.999		CtBP1KD944 P=0.011
		YFP-CtBP2(NLS)-CtBP1 P>0.006		YFP-CtBP2(NLS)-CtBP1 P=0.011		YFP-CtBP2(NLS)-CtBP1 P=0.1925		EGFP-CtBP1 P=0.1925

n, number of neurons / independent cultures analyzed
